# Haplotype-based inference of recent effective population size in modern and ancient DNA samples

**DOI:** 10.1038/s41467-023-43522-6

**Published:** 2023-12-01

**Authors:** Romain Fournier, Zoi Tsangalidou, David Reich, Pier Francesco Palamara

**Affiliations:** 1 Department of Statistics, University of Oxford, Oxford, UK; 2 Department of Genetics, Harvard Medical School, Boston, MA USA; 3 Broad Institute of Harvard and MIT, Cambridge, MA USA; 4 Department of Human Evolutionary Biology, Harvard University, Cambridge, MA USA; 5 Howard Hughes Medical Institute, Harvard Medical School, Boston, MA USA; 6 Wellcome Centre for Human Genetics, University of Oxford, Oxford, UK

**Keywords:** Population genetics, Statistical methods, Population genetics, Conservation biology

## Abstract

Individuals sharing recent ancestors are likely to co-inherit large identical-by-descent (IBD) genomic regions. The distribution of these IBD segments in a population may be used to reconstruct past demographic events such as effective population size variation, but accurate IBD detection is difficult in ancient DNA data and in underrepresented populations with limited reference data. In this work, we introduce an accurate method for inferring effective population size variation during the past ~2000 years in both modern and ancient DNA data, called HapNe. HapNe infers recent population size fluctuations using either IBD sharing (HapNe-IBD) or linkage disequilibrium (HapNe-LD), which does not require phasing and can be computed in low coverage data, including data sets with heterogeneous sampling times. HapNe shows improved accuracy in a range of simulated demographic scenarios compared to currently available methods for IBD-based and LD-based inference of recent effective population size, while requiring fewer computational resources. We apply HapNe to several modern populations from the 1,000 Genomes Project, the UK Biobank, the Allen Ancient DNA Resource, and recently published samples from Iron Age Britain, detecting multiple instances of recent effective population size variation across these groups.

## Abstract (web summary)

The authors introduce a new computational method, HapNe, for inferring the recent effective size of human populations. HapNe does not require high-quality genotype data, making it suitable for the study of ancient DNA samples.

## Introduction

The increasing availability of high-quality genomic data for both modern and ancient samples is creating exciting new opportunities for data-driven investigation of key evolutionary parameters. Among these, the effective size of a population plays an essential role in population biology^1^. A population’s effective size is defined as the number of individuals in an idealized evolutionary model^2,3^, and the ability to infer it from genomic data has a wide range of applications, including the study of past demographic events^4,5^ and cultural practices^6^, the quantification of the effectiveness of natural selection^1,7^, and the prediction of viability in conservation biology^8^.

Several statistical tools have been developed to reconstruct the trajectory of effective population size from genomic data^9^, each leveraging different genomic features and enabling the analysis of different data types. Methods that rely on the site frequency spectrum (SFS) of a sample^10–13^ avoid modeling recombination and are thus scalable, but require high-quality sequencing data to estimate the SFS and have been observed to be statistically inefficient^14^. Methods that model both mutation and recombination processes^15–19^, on the other hand, tend to scale to smaller sample sizes and require high-quality genome sequencing data. Recent approaches enable simultaneous modeling of recombination and allele frequencies in unphased sequencing data^18^, or scaling to larger sample sizes for accurately phased sequencing data^20^ and for unphased low-coverage data^21,22^. Finally, several methods that focus on capturing the signature of recombination through the sharing of identical-by-descent (IBD) haplotypes^23–27^ or linkage disequilibrium^28–31^(LD) have been developed.

Inference of recent population size fluctuations is particularly appealing because it provides unique insights into demographic and evolutionary processes that are specific to the analyzed population. IBD-based methods have been used to infer recent demographic history^23–25,27^ in SNP array and sequencing data. A key limitation of these methods is that they rely on accurate detection of IBD regions^32–35^. The performance of these algorithms depends on accurate long-range computational phasing, which may be hard to obtain, particularly in low-coverage ancient DNA data. While being a less direct measure of the signature of past recombination events, LD-based summary statistics can be computed in unphased samples, including SNP array and ancient DNA data. LD has been extensively modeled^36–40^ and applied to infer effective population size^28–31,40,41^. The most recent methods for IBD- and LD-based inference, IBDNe^27^ and GONE^31^, enable inference of population size fluctuations in time, without assuming a strictly parametrized demographic model. This strategy, however, poses additional challenges, due to the need to adequately regularize the inferred models^25,27^ to avoid reporting spurious fluctuations, while preserving manageable computational costs.

Here, we present a new method, called HapNe, that enables flexible inference of recent effective population size fluctuations using IBD or LD summary statistics, and can be used to analyze both phased and unphased SNP array or sequencing data, including low coverage or ancient DNA data with heterogeneous sampling time. Using extensive coalescent simulations, we show that HapNe accurately and efficiently infers recent demographic history, while regularizing the model to control for spurious oscillations in recent generations. We apply HapNe to reconstruct recent demographic history in both modern and ancient data, including populations from the 1000 Genomes Project and different postcodes from the U.K. Biobank data set, where we observed a bottleneck in the Late Middle Ages corresponding to the period of the Black Death. We also analyze ancient individuals from the Caribbean, Scandinavian Vikings, and individuals who lived in England during the Iron Age, observing isolation and expansion events that are consistent with past historical events, such as the transition from the Archaic to the Ceramic periods in the Caribbean.

## Results

### Overview of the HapNe algorithm

The HapNe algorithm infers recent effective population size using either IBD or LD data (see Methods and Supplementary Note for a detailed description of the algorithm). We refer to these two approaches as HapNe-IBD and HapNe-LD, respectively. HapNe-IBD uses IBD-sharing information to compute summary statistics related to the count of IBD segments of different lengths.

Briefly (see Supplementary Note for a detailed derivation, also see refs. ^23–25,27^) this approach leverages the relationship between the length and the age of IBD segments to infer the effective number of individuals that lived in a population in the past. More in detail, two individuals sharing a common ancestor may co-inherit IBD regions, which tend to be larger when the shared ancestor is recent because fewer recombination events occur in the genealogical lineages connecting these individuals. The distribution of the lengths of IBD segments thus provides information about the age of shared ancestors in a population as well as the density of common ancestors across time, which is proportional to effective population size. HapNe-IBD models these relationships using a composite likelihood that links the observed distribution of IBD segment lengths with a populations effective size trajectory.

A limitation of IBD-based inference of effective population size trajectories, however, is that it relies on accurate detection of IBD segments. This typically requires phasing information and additional modeling of haplotype sharing to differentiate between identical-by-state (IBS) and truly IBD regions. Accurate phasing and haplotype modeling may not be possible if the analyzed genomes are not of high quality or not well represented in reference panels. HapNe-LD, on the other hand, leverages summary statistics related to long-range LD (Pearson correlation between sites). These long-range correlations arise because the shared ancestors transmitting long IBD segments are typically more recent than genomic variations that are found at high frequency in the population, so that these ancestors are themselves carriers of these variants. Therefore, long-range LD is driven by the underlying presence of IBD segments, capturing demographic information used in IBD-based inference. HapNe-LD relies on a composite likelihood that models the expected distribution of IBD segment lengths and the genomic correlations they induce. These LD statistics are easy to compute and do not require genotypes to be either phased or of high quality, enabling the analysis of past demographic events in low coverage or aDNA data.

HapNe-IBD and HapNe-LD both optimize a composite likelihood. To ensure that the model is appropriately regularized, HapNe utilizes a prior on the effective population size *N*_*e*_(*t*) that favors models with minimal population size fluctuations. When the analyzed IBD or LD data does not contain sufficient signal, this regularization mechanism prevents inferring spurious variation in *N*_*e*_(*t*), which may be incorrectly interpreted as past demographic events. The resulting approximate posterior is optimized to compute a maximum-a-posteriori (MAP) estimator of *N*_*e*_(*t*) and bootstrap resampling is used to provide estimates of uncertainty through approximate 95% confidence intervals. Both methods automatically exclude genomic regions harboring unusually large amounts of IBD or LD, which may be caused by natural selection or the presence of structural variation rather than past demographic events. In addition, HapNe-LD implements a test to detect the presence of possible biases due to the presence of strong LD caused by population structure and can handle samples originating from different time points. The HapNe program is freely available as an open-source software package (see Code Availability).

### Performance on simulated modern data

**Fig. 1:**
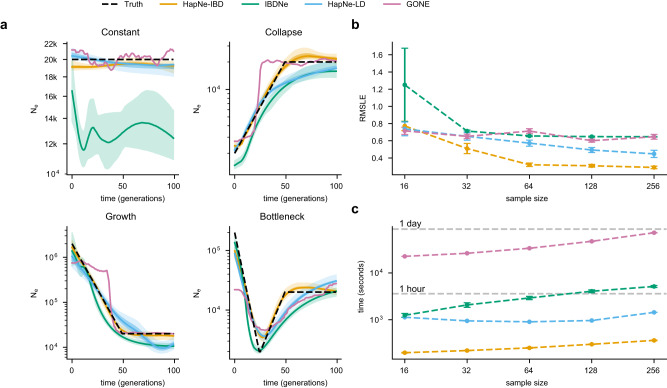
Benchmarks in simulated modern populations. **a** *N*_*e*_ estimates obtained from HapNe-IBD, IBDNe, HapNe-LD, and GONE on simulated SNP-array data (256 individuals) for four different demographic scenarios. The light and dark-shaded areas correspond to 95% and 50% confidence intervals estimated using bootstrap quantiles. **b** Accuracy of the different methods on the “Bottleneck" demographic model as a function of sample size. **c** Total running time for each method (including IBD segment detection and within-chromosome LD estimation, see Methods). In (**b** and **c**), we report the mean value across ten independent simulations as well as error bars representing 1.96 × s.e.m.

We used extensive coalescent simulations to benchmark HapNe-IBD and HapNe-LD against other recent methods for haplotype-based inference of recent effective population size. To this end, we considered several demographic scenarios (Fig. 1a, dotted black lines), including: a constant population size of *N*_*e*_(*t*) = 20,000; an exponentially expanding population with 200, 000 haploid individuals at *t* = 0 and 20,000 at *t* = 50 generations; an exponentially collapsing population with 2000 living individuals at *t* = 0 and 20,000 at *t* = 100; and a population undergoing a strong bottleneck, evolving from 200,000 haploid individuals at *t* = 0 to 2000 at *t* = 25, and then growing back to 20,000 at *t* = 50. For each of these populations, we simulated 256 diploid individuals. We generated realistic SNP-array data and used the simulated ancestral recombination graph to extract ground truth IBD segments longer than 1cM (see Methods).

We initially considered the performance of HapNe-IBD and IBDNe^33^ in an idealized setting where ground truth IBD sharing information is available (see Supplementary Fig. 1). In this scenario, HapNe-IBD generally produced lower error than IBDNe, measured using the root mean squared log-error (RMSLE) over the past 50 generations (see Methods). HapNe-IBD produced stable estimates of effective population size in the very recent past, whereas IBDNe tended to output spurious oscillations, a caveat that was highlighted by the authors^33^. We next inferred and analyzed LD summary statistics from the simulated array data using HapNe-LD. Because the LD signal reflects the presence of underlying IBD segments (see Supplementary Note), analysis of ground truth IBD data may be seen as an upper bound on the accuracy of HapNe-LD. We observed the RMSLE of HapNe-LD applied to SNP array data to be close to that of HapNe-IBD using ground truth IBD data, suggesting that HapNe-LD achieves close to optimal performance in these simulations, despite not utilizing phasing information (see Supplementary Fig. 1b). We evaluated the robustness of HapNe-LD under a misspecified recombination map and did not observe strong biases in simulations involving noisy recombination rate estimates(Supplementary Fig. 2), or when using different genetic maps in real analyses (Supplementary Fig. 3). We also tested the performance of GONE^31^, a recent LD-based method, and observed larger RMSLE in the past 50 generations (see Fig. 1b). Due to its regularization procedure, HapNe-LD tended to infer smooth changes in population size, whereas GONE inferred more rapid fluctuations (see Fig. 1a). GONE did not produce bootstrap confidence intervals in these simulations, due to an insufficient number of available SNPs (see Methods).

We next considered a more realistic scenario for the application of IBD-based methods (HapNe-IBD and IBDNe), where we inferred IBD sharing from simulated SNP array data (assuming perfect phasing, see Methods). We detected IBD sharing using the HapIBD software^35^ (see Methods); similar results were obtained by using other IBD detection methods, as well as input parameters for IBDNe (see Supplementary Fig. 4, Methods). Fig. 1a shows the output of all four methods on a data set of 256 diploid samples and results for other sample sizes are summarized in Fig. 1b (also see Supplementary Figs. 5 and 6, as well as Supplementary Fig. 7 for simulations involving larger sample sizes).

In most cases, the noise introduced by inferring IBD from the data resulted in biases in the inferred effective population sizes; IBDNe tended to underestimate recent effective population size, while HapNe-IBD tended to overestimate ancestral population size (Supplementary Fig. 5). We observed the error in IBD detection to be dependent on several factors, including demographic history, the length of the inferred segments, the software used, as well as the IBD postprocessing strategy. (see Supplementary Fig. 8).

We finally benchmarked the computational speed of these methods and observed HapNe-IBD and HapNe-LD to be more computationally efficient than IBDNe and GONE (see Fig. 1c). Computing LD scales only linearly with the number of analyzed samples, while detecting pairwise IBD sharing requires computation that is quadratic in the number of samples, making LD-based analyses more scalable. Unlike IBDNe, which requires more time to fit larger samples, HapNe-IBD only computes a fixed-size vector of the IBD segment lengths, significantly reducing computational costs for larger samples. The difference in computational time between HapNe-IBD and HapNe-LD is mainly driven by differences in the time required to compute IBD and LD summary statistics.

**Fig. 2:**
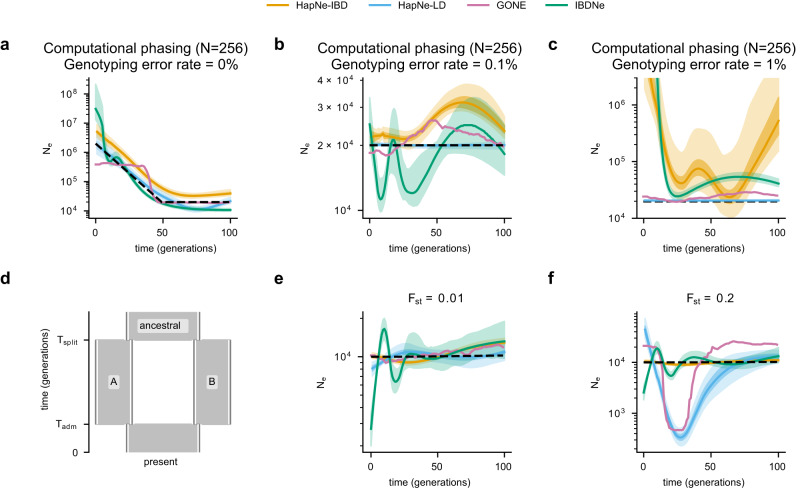
Effects of phasing, genotyping error, and population structure on population size inference. These show inference results for computationally phased genotypes from 256 simulated diploid samples *N*, using genotyping error rates of 0% in (**a**), 0.1% in (**b**), and 1% in (**c**). LD-based inference remained more robust across different demographic models and error rates. Inferred models for IBD-based methods were shifted up compared to simulations with no error (see Fig. 1). **d** This shows a demographic model used in simulations involving recent admixture. The dataset contains 100 diploid individuals sampled from a population that originates from an admixture event at time *T*_adm_ between two populations separating at time *T*_split_ (see Methods). The global effective population size (defined in Supplementary Note, Section 1.1.8) is shown using dashed black lines. Inference results for *T*_adm_ = 25 and different values of the fixation index *F*_st_ between populations *A* and *B* is shown in (**e**) and (**f**). The light and dark-shaded areas correspond to 95% and 50% confidence intervals estimated using bootstrap quantiles.

We next assessed the robustness of HapNe to the presence of haplotype phasing errors, genotyping errors, and population structure, which are often encountered in analyses of real data (see Methods). We observed HapNe-LD, which relies on unphased two-locus statistics, to be more robust than HapNe-IBD to the presence of phasing and genotyping errors. In these simulations, computational phasing reduced the sensitivity of IBD detection, resulting in an upward shift of the demographic models inferred by both HapNe-IBD and IBDNe. HapNe-LD, on the other hand, remained unaffected (Fig. 2a). We observed similar effects when genotyping errors were included (Fig. 2b, c). HapNe-LD was robust to the presence of errors, which however, caused IBD segments to break into smaller regions that led HapNe-IBD to infer spurious oscillations (see Fig. 2b, c and Supplementary Fig. 8). Finally, we investigated the impact of LD-induced by admixture (admixture LD), simulating a scenario where a population originating from a recent admixture event involving two diverged ancestral groups (see Methods, Fig. 2d). HapNe-LD estimates and removes the effects of cross-chromosome LD (see Methods), which partially corrects for the presence of population structure (Supplementary Fig. 9). This, however, does not fully account for admixture LD, which decays with genetic distance and can lead LD-based methods to infer a spurious bottleneck around the time of the admixture event (Supplementary Fig. 10). We observed the strength of this bottleneck to be proportional to the degree of differentiation between the ancestral populations. The inferred models, however, were not substantially biased for admixture events involving populations with a fixation index (*F*_st_) below 0.02 (Supplementary Fig. 11), roughly corresponding to the highest *F*_st_ observed between European populations (0.023 between Southern Italy and Finland Kuusamo), but lower than *F*_st_ observed across other groups (e.g., 0.192 between Yoruba and Japan)^42^. We, therefore, caution that HapNe-LD results may be biased in analyses of populations that experienced recent admixture events involving groups for which high *F*_st_ values are observed.

Overall, HapNe-IBD and HapNe-LD provided improved accuracy and substantially reduced computational times compared to existing methodologies. Although IBD-based inference of effective population sizes is potentially more accurate than LD-based analysis, the need to accurately detect IBD sharing is likely to introduce substantial biases in the inferred population sizes. HapNe-LD’s performance was observed to be close to that of IBD-based methods applied to ground truth IBD data and may be applied in the analysis of large sample sizes, providing several practical advantages over IBD-based methods in the analysis of real data sets.

### Performance on simulated aDNA data

**Fig. 3:**
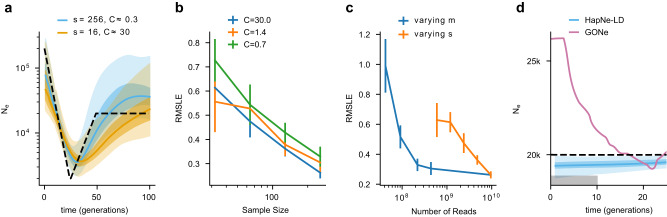
Results in simulated aDNA data. **a** HapNe-LD inference results for simulated aDNA-like data under the “Bottleneck" demographic scenario (dashed lines) where the number *s* of simulated samples and fraction *m* of missing SNPs, or equivalently the coverage *C*, are varied (see Methods). **b** RMSLE over the first 50 generations for different coverage levels. **c** Comparison of the accuracy of HapNe-LD based on two sequencing strategies. The orange line reports RMSLE for high coverage data (*m* = 0, *C* = 30) with varying sample size *s*. The blue line reports RMSLE for fixed *s* = 256 and varying coverage. **d** HapNe-LD and GONE inference results for a simulation where individuals from a population of constant size of *N*_*e*_ = 20,000 are uniformly sampled over an interval Δ*T* = 10 generations (gray shaded area). In panels **a** and **d**, the light and dark-shaded areas correspond to 95% and 50% confidence intervals estimated using bootstrap quantiles. In (**b**, **c**), we report the mean value across ten independent simulations as well as error bars representing 1.96 × s.e.m.

HapNe-LD does not require phased or high coverage data, making it especially suitable for the analysis of effective population sizes of ancient populations, where phase determination can be poor. However, analyses of aDNA data suffer from several limitations. First, analyses based on aDNA data sets tend to contain fewer samples sequenced at relatively low coverage compared with modern panels. Furthermore, different sequencing strategies balancing sample size and coverage might lead to different performances in effective population size inferences. Finally, individuals sampled at a site are unlikely to have lived at the same time, with a few notable exceptions^43,44^. If not modeled, this source of time heterogeneity may lead to biased effective size estimates. We set out to test HapNe-LD’s robustness to these sources of confounding. We first created synthetic aDNA samples by generating pseudo-haploid individuals with different levels of missingness *m*, mimicking the effects of reduced sequencing coverage *C*, with *m* ≈ *e*^−*C*^ (see Methods). We tested the relative impact of the simulated sample size *s* and coverage on HapNe-LD’s inference accuracy (see Fig. 3a and Supplementary Fig. 12 for additional demographic scenarios). As expected, RMSLE decreases when more samples are available and when coverage increases (see Fig. 3b and Supplementary Fig. 13). We then tested whether HapNe-LD would perform better when analyzing a larger number of low-coverage samples rather than a smaller number of high-coverage samples. To this end, we performed simulations where the overall number of sequencing reads is kept approximately constant, while the number of analyzed samples and their coverage are varied (see Fig. 3c and Supplementary Fig. 13). We considered an analysis involving 256 individuals and observed that reducing coverage from 30× to 1.4× had no significant impact on the performance while requiring only about 5% of the reads. Using an equivalent number of reads to perform high coverage (30×) sequencing would only allow sequencing 16 individuals, resulting in significantly higher RMSLE. These results suggest that sequencing at a coverage higher than 1–2× does not lead to significant improvements in HapNe-LD’s performance, and that HapNe-LD is more accurate when a larger number of individuals is sequenced at lower coverage compared to settings in which a smaller number of high coverage samples is analyzed, because more independent IBD segments are present in the latter case. At low sample sizes (*s* < 10), only demographic histories with a strong contraction in the recent past yielded enough signal for HapNe to infer fluctuations in our experiments (see Supplementary Fig. 14). We also examined the impact of imputing aDNA using a modern reference panel (see Methods). In these experiments, HapNe-LD obtained improved accuracy of the inferred demographic history, despite a relatively high error rate of 2.9% in the imputed aDNA genotypes (see Supplementary Fig. 15). This suggests that genotype imputation of aDNA samples may be a viable route to improve demographic inference, although additional care may be required to consider and address potential biases introduced by imputation. In these experiments, we observed that imputation induces a significant level of cross-chromosome LD, which will be flagged in HapNe’s output.

Lastly, we considered potential biases arising due to heterogeneous sampling times of the analyzed aDNA individuals. We used analytical modeling (see Methods and Supplementary Note) to confirm that, if not accounted for, heterogeneous sampling times lead to biased recent effective population size estimates. We performed simulations of aDNA samples originating from heterogeneous time locations under a constant demographic history, uniformly drawing the time offset of each sample between 0 and Δ*T* generations in the past (see Methods). In this setting, we observed that using GONE to infer effective population size leads to the spurious inference of a recent population expansion, consistent with analytical predictions under unmodeled time heterogeneity (see Fig. 3d). The HapNe-LD algorithm allows utilizing prior knowledge of sampling times (e.g., from radiocarbon dating or archeological context) in the form of a user-provided time interval for each analyzed individual (see Methods). Using simulations, we verified that this approach effectively removes recent biases due to time heterogeneity.

### Inference of recent effective population sizes in the UK Biobank and 1000 Genomes Project data sets

**Fig. 4:**
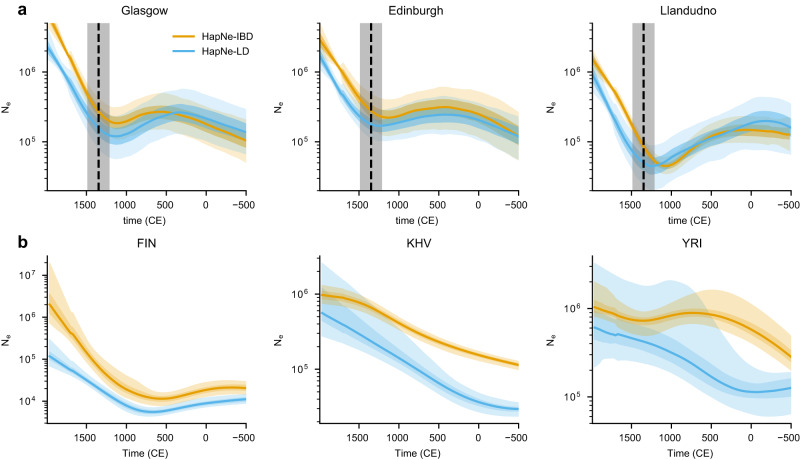
HapNe-IBD and HapNe-LD estimates of recent effective population sizes in modern populations. **a** Inference results for three postcodes: Glasgow (G), *s* = 14,724; Edinburgh (EH), *s* = 9981; and Llandudno (LL), *s* = 2089 from the UK Biobank data set. The vertical dashed line corresponds to the estimated date of the Black Death in the UK (1348, ref. ^45^). HapNe results are converted to years assuming 29 years per generation. The shaded gray area depicts how the placement of the Black Death would shift with respect to the inferred demographic models if values between 23 and 35 years per generation were assumed. **b** Inference results for three populations (Finnish, European, FIN, *s* = 99; Kinh in Ho Chi Minh City, Vietnam, South Asian, KHV, *s* = 99; Yoruba in Ibadan, Nigeria, African, YRI, *s* = 108) from the 1000 Genomes Project. The light and dark-shaded areas correspond to 95% and 50% confidence intervals estimated using bootstrap quantiles.

We used HapNe-IBD and HapNe-LD to analyze recent effective population size variation using genotype data from the UK Biobank data set (see Methods). Accurate inference of recent demographic events requires a combination of large sample sizes and small effective population sizes, which make it possible to estimate recent coalescent rates. In this case, large recent effective population sizes generally present across the UK are balanced by the large sample sizes available in the UK Biobank data set. In order to mitigate the impact of admixture LD, we focused on the larger group of samples with self-reported white British ancestry, and only considered unrelated individuals to avoid biasing demographic inference in recent generations. We grouped individuals based on the postcode of their self-reported birthplace and report analyses for three of these postcodes (see Fig. 4a, Methods). We also used FastSMC to detect IBD segments within each of these postcodes. Regions with unusually high LD or IBD sharing were excluded using HapNe’s filter (Supplementary Figs. 16 and 17).

Effective size trajectories inferred from these regions in the UK all exhibit a bottleneck event during the Late Middle Ages, which roughly corresponds to the period of the Black Death (Fig. 3a, vertical dashed line). The inferred population size for individuals from the Llandudno postcode has a significantly smaller effective population size compared to the ones inferred for Glasgow and Edinburgh. Such a smaller effective size offers a stronger source of recent demographic signal, allowing to perform inference using a smaller sample size (*s* = 2089 for Llandudno, *s* = 14,724 for Glasgow, and *s* = 9981 for Edinburgh). In contrast, detecting the more subtle contraction to a larger minimum bottleneck size in Glasgow required a substantially larger sample size, as highlighted when we downsampled data from this postcode to 2000 individuals (see Supplementary Fig. 18). In this experiment, the bottleneck was only apparent in the output of HapNe-IBD, suggesting that LD-based analysis may lead to comparably lower statistical efficiency in cases where high-quality IBD signal is available. Demographic models inferred by HapNe-IBD and HapNe-LD are broadly consistent, although HapNe-IBD tends to report a larger effective population size, with a significant shift towards more recent times. These observations are compatible with the presence of underlying IBD segments that are undetected or broken into smaller segments, due to the presence of phasing or genotyping errors in the data.

We next applied HapNe-IBD and HapNe-LD to data from the 1000 Genomes Project (1 kGP,^46^). Unlike the UK Biobank, most 1 kGP groups contain a small number of samples, which originate from large populations. Furthermore, several groups represented in the 1 kGP data set are known to have undergone recent admixture^46^. As shown in simulations, HapNe-LD is not suited for the analysis of groups with a history of recent admixture involving groups with large *F*_st_ and we, therefore, only retained a subset of populations for subsequent analysis (see Methods). We next used HapNe-LD to compute LD for each population and estimated recent IBD sharing using the HapIBD algorithm followed by a post-processing step to merge detected segments (see Methods). We then inferred recent effective population sizes using the HapNe-LD and HapNe-IBD methods and reported results for the five populations with no significant levels of CCLD and for which both methods found sufficient signal to infer demographic variation.

Figure 4b shows results for three populations meeting these criteria. Results for the other populations retained for this analysis are shown in Supplementary Fig. 19, which also includes results obtained using IBDNe. IBD-based inference consistently resulted in larger inferred effective population sizes compared to LD-based inference. As observed in simulations, this is likely caused by reduced sensitivity in the detection of IBD segments in real data, due to the presence of phasing and genotyping errors. This effect was more pronounced in 1kGP compared to the UK Biobank data set, where a larger sample size leads to higher accuracy in phasing and IBD detection. HapNe-LD infers a bottleneck at 1000 CE for the FIN population, consistent with previous reports^27,31,47^. This demographic event is inferred to have an earlier onset using IBD data, likely also a result of noisy IBD detection. Both HapNe methods suggest a recent expansion for the individuals from the Kinh population in Ho Chi Minh City, Vietnam (KHV) and for the Yoruba population in Ibadan, Nigeria (YRI). Some biases observed in our simulations are also present in the inferred demographic histories of other populations. These include population collapses in the demographic history inferred for populations with significant levels of CCLD and known recent admixture between diverged ancestral groups (Supplementary Fig. 20).

Overall, these results suggest that HapNe-LD and HapNe-IBD provide similar results when large samples and high-quality IBD data are available and suggest guidelines for the application of these tools. HapNe-LD provides more robust results than HapNe-IBD in data sets where phasing and IBD detection accuracy are reduced, at the cost of slightly reduced statistical efficiency, particularly when the presence of population structure requires the estimating cross-chromosome LD (see Supplementary Note). LD induced by recent admixture involving populations with large *F*_st_ can lead to the inference of spurious population reductions in the recent past (see Supplementary Fig. 20).

### Inference of recent demographic history in ancient populations

**Fig. 5:**
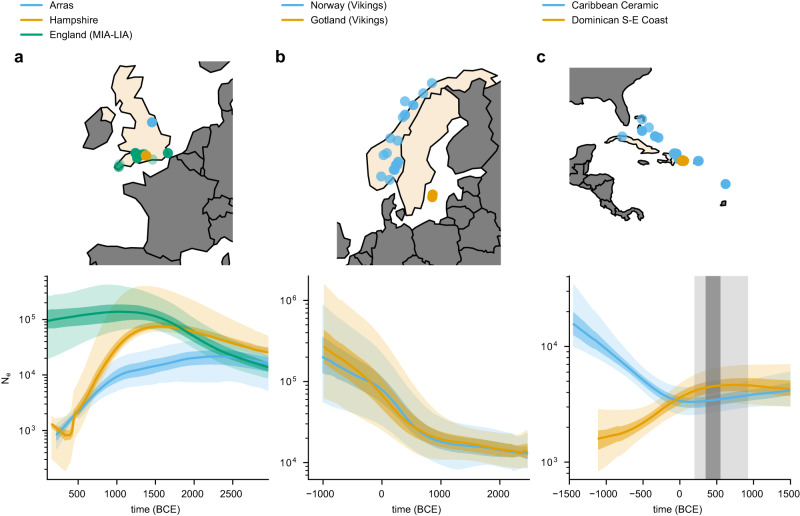
HapNe-LD estimates of recent effective population sizes in ancient populations. **a** Analysis of 49 Middle to Late Iron Age individuals from South England, compared to a subset of 14 individuals from Hampshire, and to 24 individuals related to the Arras culture near Yorkshire. **b** Inference based on 22 Viking samples found in modern Norway (blue) and 28 found in Gotland, a Swedish island (red). **c** Effective population size inference based on 71 unrelated individuals from the Caribbean Ceramic clade and 18 from the Dominican South-East coast subclade. The dark-gray shaded area corresponds to the estimated date for the transition from the Archaic to Ceramic culture in the region. The light and dark-colored shaded areas correspond to 95% and 50% confidence intervals estimated using bootstrap quantiles. The light gray-shaded area depicts how the placement of this transition would shift with respect to the inferred demographic models if values between 25 and 35 years per generation were assumed. The dots on the maps represent the location of the samples. The figure was made with Natural Earth. Free vector and raster map data @ naturalearthdata.com.

We applied the HapNe-LD method to aDNA sampled from four different sites for which large cohorts from similar time strata were available (see Methods and Supplementary Tables 1–8). We excluded individuals for which known close relatives were present in the data set (see Methods), which would otherwise lead to a smaller inferred effective population size. It is still possible that undetected distant relationships remain present in these groups; if present, our analyses interpret these more distant relationships as reflecting demographic phenomena, rather than sample ascertainment. We first analyzed a group of recently published individuals excavated in Pocklington, Yorkshire, UK^48^ (see Fig. 5a). The archeological context suggests that this group belongs to the Arras culture, which is distinctive relative to other Iron Age cultures in the UK but shows similarities with contemporary cultures in the Paris Basin and Ardennes/Champagne regions of France. These individuals were found to be unusually highly drifted from nearby groups, although their F-statistics do not highlight significantly divergent admixture histories. This suggests that these groups share common origins but may have been isolated for some time or that they originated from a later migration event^48^. This hypothesis is compatible with the effective population sizes we observed when running HapNe on 24 individuals from the Arras culture, which we compared with 49 individuals from South England (Supplementary Tables 2–4). Because the Arras are sampled from a smaller geographic region compared to the samples from South England, we also considered a subset of 14 individuals from South England sampled from a similarly localized region in Hampshire.

Both the Arras and the Hampshire groups displayed a significant recent population contraction, probably reflecting geographic localization. However, the demographic model inferred for the Hampshire samples grows back to the population size inferred for South England, whereas the size observed for the Arras samples remains smaller. This difference in population sizes is consistent with the recent observation of high genetic drift between the Arras and other groups in these regions, possibly reflecting isolation or distinct origins^48^. Cross-chromosome LD for these groups was found to be negligible, suggesting that the observed demographic signature is not due to population structure (see Supplementary Table 1), although recent admixture might create a similar collapse (see Supplementary Fig. 11). The small population size of the Arras group might also explain why this population was found to be unusually highly drifted from nearby groups. The recent effective population size inferred for individuals in the South of England was compatible with population size estimates obtained for modern UK Biobank individuals, although confidence intervals were large over the first 1000 years due to a reduced sample size.

We next analyzed 22 genetically similar individuals from the Viking Age buried in Norway, together with 28 individuals from the south-east Swedish island of Gotland^43^ (Fig. 5b and Supplementary Tables 5 and 6). Norwegian and Swedish Vikings have been observed to have a slightly smaller proportion of ancestry from Neolithic farmers from Anatolia compared to Vikings from Gotland. On the other hand, Vikings from Gotland have a relatively higher estimated fraction of ancestry shared with Bronze Age individuals from the Baltic region. Despite these differences, the demographic histories inferred by HapNe-LD for the recent past of these individuals substantially overlap, and both trajectories show a significant expansion during the iron age (−500 to 800 CE).

Finally, we focused on 71 unrelated individuals from the Caribbean, first analyzed in ref. ^49^ (*n* = 62) and ref. ^50^ (*n* = 9) spanning ~1149 to ~1440 CE (Supplementary Tables 7 and 8). For these samples, HapNe-LD infers a weak sign of a bottleneck occurring around 1 CE, followed by a significant expansion, as shown in Fig. 5c (blue line). This pattern may reflect the transition from the Archaic to Ceramic context about 2500–2300 years ago (Fig. 5a, gray area), which has been associated with migration events in the region^49^. We also extracted and separately analyzed a subgroup of individuals from South-East Dominican sites (Fig. 5c, red). These individuals are part of a subclade previously identified in ref. ^49^. The population size inferred for this group matches that of the broader Caribbean group in the deep past, consistent with common origins, but shows a distinctive sign of contraction in the more recent past. LD induced by population structure is detectable in these individuals, which may partially explain the observed contraction (Supplementary Table 1). Nevertheless, the sizes inferred by HapNe-LD in the recent past roughly match those inferred using runs of homozygosity^51^, supporting the possibility of a population contraction starting after the transition from the Archaic to the Ceramic period^49^. As in the case of the Arras and Southern England individuals, these demographic patterns may also be due to isolation by distance, where samples originating from different islands result in a larger effective size when considered together.

## Discussion

We developed an algorithm, called HapNe, that leverages the count of IBD segments of different lengths (HapNe-IBD) or long-range LD (HapNe-LD) to infer recent effective population size fluctuations in modern or ancient DNA data. HapNe-IBD and HapNe-LD implement a number of preprocessing steps, as well as tests to verify that sufficient recent demographic signal is present in the data and to detect the presence of admixture LD. Both methods minimize a power-likelihood based on an analytic link between observed summary statistics and the effective population size and use regularization to avoid producing spurious oscillations. We used extensive simulation to show that both HapNe methods were more accurate and computationally faster than available algorithms for IBD-based and LD-based inference of recent demographic history, producing lower error and fewer spurious oscillations. These simulations also showed that while HapNe-LD does not require high-quality or phased data and scales better with sample size, its performance can be close to that of IBD-based methods applied to ground truth IBD information. Finally, we applied HapNe to several modern and aDNA data sets, detecting evidence for recent past demographic events across these populations. These include population size contractions corresponding to the period of the Black Death in different regions of the UK, as well as bottleneck and expansion events in 1000 Genome Project populations. In aDNA data, these analyses provided evidence for divergence and isolation events, as well as shared demographic histories in subgroups from several ancient populations with diverse geographic and temporal origins.

Our analyses suggest that LD-based inference of recent demographic variation provides a route to circumenting biases that may arise in IBD-based demographic inference. Although the spectrum of shared IBD haplotypes is an effective source of information for analyses of past demographic events, accurately estimating IBD sharing is complicated in low coverage and aDNA data and may lead to biased results. This may also be the case in modern populations when limited data availability prevents accurate phase estimation. Although summary statistics of LD rely on less direct observation of historical recombination events, they may be effectively computed in unphased and low-coverage data sets. This enables analyzing recent demographic events in samples from poorly represented populations and, coupled with modeling of heterogeneous sampling time, in aDNA data sets. Performing both IBD-based and LD-based analyses may offer validation for an inferred demographic model and allow testing for the presence of biases in either approach. An additional source of potential bias in methods for demographic inference is linked to the need to make assumptions about the type of demographic model being inferred. In this context, approaches that avoid relying on a predefined set of models provide more flexibility, but require further tuning strategies to balance the desired sensitivity to past demographic events with the need to prevent the inference of spurious fluctuations. Our work suggests that the use of self-tuning regularization mechanisms helps mitigate the risk of spurious inferred fluctuations. Finally, our analyses highlight the importance of accurately preprocessing both IBD and LD signals before performing demographic inference, as results may vary significantly if unfiltered data is utilized. Key preprocessing steps include testing for the presence of admixture LD and systematically filtering out regions of the genome that harbor unusually high IBD sharing or LD (see e.g., Supplementary Fig. 16). These may be due to natural selection or the presence of structural variation and lead to biases in analyses of demographic history and selection if not accounted for.

We outline several limitations and directions of future development for this work. First, HapNe-LD assumes that the LD signal observed in the data is solely due to past population size fluctuations. In some instances, residual admixture LD can be present in the data after filtering, causing a spurious contraction in the recent past and creating the need to carefully interpret models that resemble this type of signature. In general, a contraction in the recent past might be due to a decrease in the census size of the population, isolation, or an artifact of recent admixture. Similarly, HapNe-IBD currently only relies on the observed spectrum of IBD sharing, which may be biased due to inaccurate IBD detection. Future work may allow explicit modeling of type-1 and type-2 errors in IBD detection, mitigating biases in the inferred demographic models. Since the HapNe-LD and HapNe-IBD methods are subject to different sources of bias, their output can be compared to check the validity of their output. Second, while regularization helps prevent the inference of spurious demographic fluctuation, it leads to favoring constant and exponential demographic histories that lack fluctuations if these are not supported by the data. When interpreting demographic models inferred by HapNe, it is important to note that an inferred constant growth rate may reflect insufficient evidence for past demographic variation (see e.g., Supplementary Fig. 18), which causes the model to be more strongly regularized. For the same reason, when the data contain limited demographic information, HapNe may produce tight bootstrap confidence intervals for demographic histories that are closer to constant population size. These should also not be interpreted as strong evidence for a constant size, but as a lack of evidence for past fluctuations. Third, we note that both LD-based and IBD-based analyses rely on preprocessing steps to filter out genomic regions that may otherwise lead to biases. These excluded regions harbor unusually high or low density of LD or IBD signal, which may be caused by non-demographic factors such as natural selection or the underlying presence of structural variation^52^ (see Supplementary Fig. 17). HapNe-LD and HapNe-IBD automatically detect and remove these genomic regions. This is currently achieved by dropping entire chromosome arms but a less conservative and likely more computationally intensive approach may be devised. Fourth, HapNe-LD currently corrects for time heterogeneity by marginalizing out the time difference between the samples included in the analysis. Future extensions may allow HapNe to increase inference accuracy by correcting the LD for each pair of samples separately.

Finally, HapNe-LD makes several model simplifications, including the assumption that the analyzed samples come from a single population. A promising direction for future work is to extend HapNe to allow it to explicitly account for multiple populations and infer coalescent rates across groups, improving the analysis of more complex demographic models such as those involving isolation by distance, divergence, and admixture. Similarly, HapNe-LD is currently focused on the inference of recent demographic history, but may be extended to the analysis of deeper time scales by modeling variation in allele frequencies, which are currently assumed to be constant in time. Despite these limitations, we expect that the HapNe framework developed in this work will offer valuable insights into past demographic events in both modern and ancient DNA data.

## Methods

### Simulated genetic data

We used the ARGON simulator^53^ (version 0.1.160415) to generate synthetic genotypes and ground truth IBD data for modern and ancient populations. Simulations with time heterogeneity were performed using msprime^54^ (version 1.1.1). We simulated genomes of 36.23 Morgans, split into 39 independent regions corresponding to human chromosome arms. We used a mutation rate of *μ* = 1.65 × 10^−8^ and a recombination rate of *ρ* = 1 × 10^−8^ per generation per base pair, except in experiments where human genetic maps were used. To simulate SNP data, we then downsampled sequencing data to match the genotype density and allele frequency spectrum observed using Chromosome 2 of the UK Biobank data set, using 50 evenly spaced MAF bins. We generated unphased diploid individuals by randomly pairing simulated haplotypes. Ancient data was generated using a similar procedure, downsampling to 1240k SNP array densities. We also added two additional steps to simulate low-coverage data. We first transformed the data into pseudo-haploid individuals by randomly sampling one haplotype at each site. We then set each site as missing with probability *m*, related to a simulated coverage parameter *C* through the relationship *m* ≈ *e*^−*C*^, further described below.

In the simulations that incorporate admixture, we employed a demographic history starting with an ancestral population of size *N*_*e*_ = 10, 000 which splits into two populations, A and B, at time *t* = *t*_split_. Populations A and B then evolve independently, each with an effective population size of *N*_*e*_ = 5000. At time *t* = *t*_adm_, the two populations merge into a single population, with an equal contribution. The newly created population has a size of *N*_*e*_ = 10, 000. It then experiences one of four scenarios (Supplementary Fig. 11). In one scenario, the population remains constant until *t* = 0. In the other scenarios, the population undergoes an exponential expansion with *N*_*e*_ = 100, 000 at *t* = 0, an exponential collapse with *N*_*e*_ = 1000 at *t* = 0, or a bottleneck with *N*_*e*_ exponentially decaying to 1, 000 at *t* = *t*_adm_/2 before exponentially growing back to 10, 000 at *t* = 0. For convenience, we report the value of *F*_st_ instead of *t*_split_ when discussing the results; the link between these two quantities is found described in Supplementary Note Section 1.1.9.

### Simulation of missingness and coverage

We simulated low coverage data by discarding a proportion *m* of the SNPs of each individual, but often report results referring to corresponding sequencing coverage parameters. To this end, we assumed a simple model where a genome of length *G* is sequenced using *N* reads of length *L*. Using this notation, the probability that a randomly selected site along the genome is not spanned by a read is:

1 $$m={\left(1-\frac{L}{G}\right)}^{N}\\={\left(1-\frac{C}{N}\right)}^{N}\\ \approx \, \, {e}^{-C},$$

where $C\equiv \frac{NL}{G}$ represents the coverage parameter.

This relation can also be used to obtain a link between *m* and the number of reads:

2 $$N=-s\frac{\log (m)}{z},$$

where $z=-\log (1-\frac{L}{G}) \, > \, 0$ and *s* is the number of sampled individuals with missingness *m*.

### Imputation of simulated ancient data

To simulate imputed aDNA data, we simulated 32 ancient samples from 30 generations before present as well as 200 modern individuals, using msprime^54^ (v 1.1.1). These individuals were sampled from a demographic model with an ancestral population size of 20,000 haploid individuals, which undergoes an exponential expansion starting at 80 generations before present. The expansion makes the population reach 2,000,000 individuals at generation 30, after which it maintains a constant size until present day. We downsampled the simulated 32 ancient individuals to match 1240k SNP array mutation and allele frequency densities. We simulated short reads (150bp) at the target coverage on the array SNPs. These reads were written to SAM file format and compressed into BAM file format using samtools (version 1.13). We further simulated a reference fasta file using msprime reference allele at all polymorphic positions and then aligned the reads to the reference sequence and called genotype likelihoods using BCFtools (version 1.15.1). We then used GLIMPSE (version 1.1.1) to impute the ancient data, leveraging the called genotype likelihoods, using the 200 modern simulated samples as a reference panel, assuming perfect phasing and 0 genotyping error. This imputation process results in a genotype call being made for every variant that is polymorphic in the reference panel or the ancient samples. We reported imputation *r*^2^, the squared correlations between the imputed allele dosage and the ground-truth sequencing data for the ancient samples across the polymorphic variants (Supplementary Fig. 15). Imputation quality at coverage of 1× was above 90% for SNPs with MAF above 25%, used in our analyses.

### Computation of LD

We consider a panel of *s* individuals, *M* sites and genotypes ${\tilde{G}}_{i,x}\, \sim \,{{{{{{{\rm{Bin}}}}}}}}(2,\,{p}_{x})$ for individual *i* at site *x* with minor allele frequency *p*_*x*_. We first standardize the genotypes by computing ${G}_{i,x}=\frac{{\tilde{G}}_{i,x}-2{\hat{p}}_{x}}{\sqrt{2{\hat{p}}_{x}(1-{\hat{p}}_{x})}}$, where ${\hat{p}}_{x}$ is the estimated allele frequency. The LD between two sites *x* and *y* is computed as the *R*^2^ statistic:

3 $${R}_{x,y}^{2}=\frac{{\left({\sum }_{i=1}^{s}{G}_{i,x}{G}_{i,y}\right)}^{2}-\left({\sum }_{i=1}^{s}{G}_{i,x}^{2}{G}_{i,y}^{2}\right)}{s(s-1)}.$$

The computation of this statistic scales linearly with the number of samples (${{O(s)}}$). Note that this estimator is biased due to the use of ${\hat{p}}_{x}$ instead of the unknown allele frequency *p*_*x*_ during the normalization step. We describe a procedure used at runtime to debias these estimates in the Supplementary Note. The LD of pseudo-diploid individuals is computed using the same approach, with $\frac{1}{2}{\tilde{G}}_{i,x}\, \sim \,{{{{{{{\rm{Bin}}}}}}}}(1,\,{p}_{x})$.

### Detection of IBD segments

We used FastSMC^34^ (version 1.2), HapIBD^35^ and RefinedIBD^33^ (version 17jan20) to detect IBD segments in simulated and real data analyses. HapIBD and RefinedIBD were used with recommended parameters for SNP-array data (default parameters). We ran FastSMC using parameters min *m* = 0.5 (minimum cM length) and *t* = 100 (IBD time threshold). Decoding quantities were generated based on 30 samples using a European demographic history. FastSMC was run using multiple jobs, so that each job considers at most 100 haploid samples. The IBD segments inferred by HapIBD and RefinedIBD were post-processed using the merge-ibd-segments tool (see URLs), using the default parameters. We observed that this post-processing step improves the accuracy of the inferred IBD segment length distribution when genotyping and sequencing errors are present in the data. In these scenarios, FastSMC may break segments into shorter regions based on their estimated posterior probability. However, a post-processing tool to merge these fragmented regions and improve the accuracy of segment length estimates is not available for FastSMC, so we used HapIBD and RefinedIBD for most analyses, except for the use of FastSMC in the analysis of the UK Biobank data^34^, which is accurately phased.

### HapNe-IBD and HapNe-LD algorithms

We developed two algorithms to infer recent effective population size fluctuations *N*_*e*_(*t*) from a set of *s* samples, called HapNe-IBD and HapNe-LD. Both approaches take summary statistics {*Y*_*i*,*b*_} as input and maximize a pseudo-posterior function for *N*_*e*_(*t*). The input data set {*Y*_*i*,*b*_} is split into 39 genomic regions corresponding to chromosome arms indexed by *i*, using 0.5 cM long bins indexed by *b*.

HapNe-IBD takes as input a list of IBD segments of length $N \sim O({s}^{2})$. Input data {*Y*_*i*,*b*_} corresponds to the count of IBD segments in region *i* whose length lies in bin *b*. Bins start at 2cM and end at the largest detected IBD segment. We assume that each of these counts is the realization of a Poisson random variable, with demographic-dependent mean parameter ${\mu }_{b}\left({N}_{e}(t)\right){L}_{i}$, where *L*_*i*_ is the length of the *i*th region (${\mu }_{b}\left({N}_{e}(t)\right)$ is described in the Supplementary Note). To handle overdispersion, we used a quasi-likelihood approach to compute a weight parameter ${\phi }_{b}^{2}$ that multiplies the variance in each bin.

HapNe-LD uses average *R*^2^ statistics as input data {*Y*_*i*,*b*_}. This input is computed in $O(sm)$, where *m* is the total number of loci. We assumed that these observations are realizations of a Normal random variable, with a distance-dependent mean parameter ${\mu }_{b}\left({N}_{e}(t)\right)$ (see Supplementary Note for a detailed description of ${\mu }_{b}\left({N}_{e}(t)\right)$). The variance parameters ${\phi }_{b}^{2}$ were estimated using the usual variance estimator within each bin.

Give a set of IBD or LD observations {*Y*_*i*,*b*_} for the *i*th genomic region and *b*th bin, HapNe aims to maximize *P*(*N*_*e*_(*t*)∣{*Y*_*i*,*b*_}) under the following assumptions. First, *N*_*e*_(*t*) is a piece-wise exponential function from *t* = 0 to *t* = *t*_*max*_ generations, and remains constant afterwards. In all our analyses, we used *t*_*max*_ = 125 generations. The lengths of the time intervals are iteratively tuned so that each time interval contains the same number of expected ancestors of IBD segments (see Supplementary Note). Second, we assume that there exists a prior on the effective population size ${p}_{{N}_{e}}(\theta )$, where *θ* represents the set of parameters defining *N*_*e*_(*t*). A discussion about the choice of this prior can be found in the Supplementary Note. Third, we assume that the total likelihood *P*({*Y*_*i*,*b*_}∣*N*_*e*_) can be approximated by a power likelihood^55,56^ and be written as $P(\{{Y}_{i,b}\})={\prod }_{i,b}P{({Y}_{i,b})}^{c}$. If we assume that bins on different chromosomes are not correlated, the exponent *c* captures the correlations between the bins of a region. When there is no correlation in the data, *c* = 1 recovers the true likelihood, whereas if all bins are fully correlated, setting $c=\frac{1}{{n}_{{{{{{{{\rm{bins}}}}}}}}}}$ leads to the likelihood of a single observation. We discuss how this hyperparameter is automatically tuned using a heuristic model selection rule in the Supplementary Note.

Once the time intervals and the value of the regularization parameter are fixed, HapNe assesses the uncertainty of the prediction by performing 100 bootstrap iterations. For each iteration, HapNe samples chromosome arms with replacement to create new input data, and estimates the effective population size. The 2.5th, 25th, 75th, and 97.5th percentiles are reported at each generation to obtain 50% and 95% confidence intervals.

### Comparisons to other methods

To perform method comparisons, we simulated genotypes based on the demographic models shown in Fig. 1 and used the methodology described above to compute summary statistics. We ran HapNe-IBD, HapNe-LD, IBDNe (version 23Apr20.ae9), and GONE (retrieved Jun 22, 2021). We used default parameters for all methods, except for IBDNe where we set gmin=1 in simulated data, as recently recommended^57^. The simulated SNP array data did not contain enough sites to perform the SNP bootstrapping strategy used by GONE to produce confidence intervals in sequencing data. All computations were run on an Intel Skylake 2.6 GHz architecture on the Oxford Biomedical Research Computing cluster.

We reported the root mean squared log-error (RMSLE) over the first 50 generations as a measure of accuracy. If *N*_*e*_(*t*) and ${\hat{N}}_{e}(t)$ denote the true and predicted demographic models, the accuracy is defined as:

4 $${{{{{{{\rm{RMSLE}}}}}}}}=\sqrt{\frac{1}{50}\mathop{\sum }\limits_{{t}_{i}=1}^{50}{\left(\log \left({\hat{N}}_{e}({t}_{i})\right)-\log \left({N}_{e}({t}_{i})\right)\right)}^{2}}$$

We performed ten independent sets of simulations and computed error bars reported in each plot as 1.96 × s.e.m.

### Filtering of high IBD and LD regions

To mitigate the impact of natural selection and structural variation, HapNe applies a filtering algorithm to exclude chromosome arms with unusual amounts of IBD sharing or LD. For LD data, parameters of a normal distribution are computed for each bin using the median and quantiles of the observed data. We used this quantile-based approach instead of moment-based estimators so that the inference is robust in the presence of the outlier regions we aim to filter out. Then, each genomic region is discarded using the following two heuristic rules. First, the deviation between the observed LD in the region and the median must be within 6 standard deviations. Second, the observed values must cross the median at least once, i.e., a region cannot have all its observations above or below the median. The IBD data is filtered using a similar approach. For each region, the mean of the Poisson distribution and the dispersion factors are computed for each bin using all others regions. The region is discarded if the sum of its squared deviance residuals is in the upper or lower *α*-quantile of the underlying *χ*^2^ distribution, with *α* = 10^−12^. The procedure is performed a second time, without considering the discarded regions, to prevent outliers to impact the final result.

### LD-based test for population structure

Population structure creates long-range LD between unlinked pair of sites. HapNe allows testing for LD due to population structure by computing cross-chromosome LD (CCLD). In the absence of CCLD, we expect the correlations between two sites *x* and *y* located on different chromosomes to be only due to finite sample sizes (see Supplementary Note):

5 $${\mathbb{E}}\left[{G}_{i,x}{G}_{i,y}{G}_{j,x}{G}_{j,y}-\frac{4}{({N}_{x}-1)({N}_{y}-1)}\right]=0,$$

where *N*_*x*_ and *N*_*y*_ are the number of observed haplotypes on sites *x* and *y*, respectively. Because the LD is only computed between pairs of sites containing at least two overlapping observations, *N*_*x*_ and *N*_*y*_ are not independent variables. HapNe-LD computes the empirical mean of Eq. (5) for each pair of chromosomes and then performs a *t*-test to check for deviation from the 0-mean hypothesis.

### Time heterogeneity in the set of analyzed samples

Most aDNA data sets contain samples originating from different time points, with an estimated date range spanning many generations when the archeological context is used to date the samples. We thus extended HapNe-LD to account for time heterogeneity and uncertainty. The user can provide a date range for each sample. This information is used by HapNe to compute the density of the ages of a randomly selected pair of individuals. This density is then used to marginalize out the age of the oldest sample and the generation gap between the two individuals under the SMC approximation, resulting in an unbiased estimator of the effective population size (see Supplementary Note).

### Inference of demographic history in the UK Biobank

We analyzed the subset of 305,784 unrelated samples with self-reported White British ancestry, corresponding to the individuals reported in ref. ^58^ that did not withdraw from the study and whose birth location can be assigned to a postcode in the U.K. (13,995 were removed because of this last condition). We focused on 727,103 genotyped autosomal variants, which we phased using Beagle 5.1^59^. We then grouped the individuals based on their self-reported birth location, labeling each of them with the first 1 or 2 letters of their corresponding postcode. We randomly picked postcodes with different sample sizes to infer population sizes. LD computations and IBD detection steps were performed using the procedure described above, using genetic maps corresponding to the GRCh37 genome build.

### Inference of Demographic history in the 1000 Genomes Project

We downloaded the *N* = 2504 high-coverage sequenced samples from the 1000 Genomes Project data set^60^. The samples were grouped according to 26 population labels. For the IBD detection step, we downsampled the sequenced data to match the density of SNP array variants found in the UK Biobank, using the procedure described above. IBD segments were inferred using HapIBD and merged using the post-processing tool described above. HapNe-LD only utilized variants with MAF > 0.25, as in previous analyses (see Supplementary Note). Populations flagged as having unusual cross-chromosome LD or no signal were excluded from the analysis (Supplementary Fig. 20). Genetic maps corresponding to the GRCh38 genome build were used for this analysis.

### Inference of demographic history in ancient data

We downloaded version 50.0 of the Allen Ancient DNA Resource (AADR) dataset^61,62^. For each analysis, we started by removing related individuals reported in the annotation files present in the dataset. For each family, the individual with the highest coverage was kept. Information about sample ages was also extracted from the annotation file and used as input for HapNe-LD. To ensure high data quality, we filtered the datasets to include only variants and individuals with a missing data rate *m* of less than 80%, corresponding to a coverage of approximately 0.22×. Specific information about each population is present in Supplementary Tables 1–8.

### Software

We used ARGON^53^ (version 0.1.160415) and msprime^54^ (version 1.1.1) to simulate synthetic data. We used Plink 1.9^63^ and Plink 2.0^64^ to preprocess genetic files. We used FastSMC^34^ (version 1.2), HapIBD^35^ (version 1.0, 23Apr20.f1a), and RefinedIBD^33^ (version 17jan20) to detect IBD segments in simulated and real data analyses. We used IBDNe^27^ (version 23Apr20.ae9), and GONE^31^ (retrieved on Jun 22, 2021) to infer effective population sizes. Data processing and plotting were performed using Numpy (1.23.4)^65^, Pandas (1.5.1)^66^, SciPy (1.9.3)^67^, Numba (0.56.3)^68^, Matplotlib (3.4.3)^69^, Seaborn (0.12.2)^70^, and Geopandas (0.12.2)^71^.

### Reporting summary

Further information on research design is available in the Nature Portfolio Reporting Summary linked to this article.

### Supplementary information

Supplementary Information

Reporting Summary

## Data Availability

All data sets used for this study can be accessed using the following links. The data sets simulated for this study are available at 10.5281/zenodo.10024899. UK Biobank data can be accessed by approved researchers through http://www.ukbiobank.ac.uk/. Other data sets can be downloaded from the following URLs: genetic maps https://ftp-trace.ncbi.nih.gov/1000genomes/ftp/technical/working/20110106_recombination_hotspots/, 1000 Genomes Project phase three^60^https://www.internationalgenome.org/data/and the Allen Ancient DNA Resource^61^https://reich.hms.harvard.edu/allen-ancient-dna-resource-aadr-downloadable-genotypes-present-day-and-ancient-dna-data(Supplementary Tables 1–8).

## Code availability

The HapNe software package is freely available at https://palamaralab.github.io/software/hapne.
