# Supplementary material for: Haplotype-based inference of recent effective population size in modern and ancient DNA samples
Source: Nat Commun. 2023 Dec 1;14:7945. doi: 10.1038/s41467-023-43522-6 (PMC10692198; doi:10.1038/s41467-023-43522-6)
Supplement: Supplementary file 1 — Supplementary Information [file 41467_2023_43522_MOESM1_ESM.pdf]

Supplementary Information

Haplotype-based inference of recent effective population size in modern and ancient DNA samples

Fournier et al.

1 Supplementary Note

1.1 Derivation of the IBD and LD models

This note describes the models used to infer effective population size from IBD and LD summary statistics. We first describe a link between the effective population size and the probability that two sites are spanned by an IBD segment under the SMC' model[1], as well as computationally tractable approximations used in several derivations. Related work on calculations presented in this section may be found in[2–11]. We then provide details on how these models are used to perform inference based on IBD and LD summary statistics. We conclude by describing further details of the LD model related to low coverage data, time-heterogeneity, and population structure induced LD.

1.1.1 Notation

We aim to infer the effective population size $N_e(t)$ based on the genotype of s samples consisting of m markers. For simplicity, we will assume that t is a continuous variable, with $t = 1$ corresponding to 1 generation. Note that $N_e(t)$ refers to haploid individuals in the population. Although $N_e(t)$ is the quantity of interest, we will derive several expressions in terms of its inverse $\gamma(t) \equiv \frac{1}{N_e(t)}$, the coalescent rate, as well as the cumulative coalescent rate $\Gamma(t) \equiv \int_0^t \gamma(v)dv$.

1.1.2 Survival function for a change of ancestor

Using the above notation, the distribution of the age of the most recent common ancestor (TMRCA) of a pair of haplotypes under the coalescent[12] may be expressed as:

$$f(t) = \gamma(t)e^{-\Gamma(t)}, \tag{1}$$

which for a constant coalescent rate takes the form of an exponential waiting time $f(t) = \gamma e^{-\gamma t}$, leading to $\mathbb{E}[T] = N_e$.

Given the MRCA at site x, with TMRCA$= t$, we are interested in the genetic distance U at which a change of ancestor is observed. This requires a recombination event, which occurs at rate $2t$ (see e.g.[13]). When a recombination event happens, a new lineage is created at a time $V \sim \text{Uniform}(0, t)$. This new lineage will not lead to a change of ancestor if it coalesces back to the lineage from which it branched out between V and t. We refer to this kind of coalescent event

as a "healing" event and denote its probability by $p_h(t)$. To derive an expression for $p_h(t)$, we note that the coalescent rate of the new lineage is given by $f_2(t) = 2\gamma(t)e^{-2\Gamma(t)}$, with a factor 2 appearing because the new lineage can coalesce with either of two original ones. Healing requires the new lineage to coalesce between v and t, which happens with probability $\frac{\int_v^t f_2(w)dw}{1-\int_0^v f_2(w)dw}$. It also requires the new lineage to coalesce to the original lineage, which happens with probability $\frac{1}{2}$. Together, these terms lead to the following expression, also derived in[7]:

$$\begin{aligned} p_h(t) &= \frac{1}{t}\int_0^t \frac{1}{2}\frac{\int_v^t f_2(w)dw}{1-\int_0^v f_2(w)dw}dv \\ &= \frac{1}{2} - \frac{e^{-2\Gamma(t)}}{2t}\int_0^t e^{2\Gamma(v)}dv \end{aligned} \tag{2}$$

For a constant demographic history with coalescent rate γ, this becomes:

$$p_h(t) = \left(\frac{1}{2} + \frac{e^{-2\gamma t}-1}{4\gamma t}\right), \tag{3}$$

Thus, the waiting distance for a change of ancestor is exponentially distributed with rate $2t(1-p_h(t))$ and its survival function is given by:

$$S(u|t) = e^{-2tu(1-p_h(t))} \tag{4}$$

We obtain $S(u)$ by marginalizing the TMRCA,

$$S(u) = \int_0^\infty e^{-2tu(1-p_h(t))}f(t)dt \tag{5}$$

For a constant population size, this expression becomes:

$$S(u \mid \gamma) = 2^{\frac{1}{2}\left(\frac{u}{\gamma}-1\right)}e^{-\frac{u}{2\gamma}}\left(-\frac{u}{\gamma}\right)^{-\frac{\gamma+u}{2\gamma}}\left(\Gamma_{Euler}\left(\frac{u+\gamma}{2\gamma},0\right) - \Gamma_{Euler}\left(\frac{u+\gamma}{2\gamma},-\frac{u}{2\gamma}\right)\right), \tag{6}$$

where Γ_{Euler} denotes the incomplete Euler gamma function $\Gamma_{Euler}(z,a) = \int_a^\infty e^{-t}t^{z-1}dt$. This survival function, also derived in[14], assumes an underlying SMC' model[1], but does not lead to a closed-form solution when a piece-wise constant function $\gamma(t)$ is utilized. To obtain a tractable

expression, we introduce an approximation of the SMC' model. Using a Taylor expansion, Eq. 4 may be written in the form:

$$
\begin{aligned}
S(u \mid t) &= e^{-2t\left(1-p_h(t)\right)u} \\
&= e^{-2tu}\left(1+\sum_{k=1}^{\infty}\frac{(p_h(t)2tu)^k}{k!}\right) \\
&= e^{-2tu}\left(1+\sum_{k=1}^{\infty}p_h^k(t)\frac{\int_0^u (2t)^k v^{k-1}e^{-2tv}e^{2tv}dv}{(k-1)!}\right) \\
&= e^{-2ut}+\sum_{k=1}^{\infty}p_h^k(t)\int_0^u f_{erl}(v;2t,k)e^{-2t(u-v)}dv,
\end{aligned}
\tag{7}
$$

where $f_{erl}(v;2t,k)=\frac{(2t)^k v^{k-1}e^{-2tv}}{(k-1)!}$ is the probability density function of the sum of k exponential random variables with rate $2t$. In the last sum, k can be interpreted as the number of healing events observed within a distance u. The SMC approximation, where each recombination event leads to a change of ancestor[15], is recovered by only considering the first term and discarding the sum:

$$S_0(u \mid t) = e^{-2tu}. \tag{8}$$

For a constant demographic history, the survival function becomes:

$$S_0(u \mid \gamma) = \frac{\gamma}{\gamma+2u}. \tag{9}$$

Note that this recovers the expression derived in [16] using a different approach. This approximation may become poor when working with small populations and short genetic distances. For example, considering $u = 1\text{cM}$ and $\gamma = \frac{1}{1{,}000}$ leads to a relative error $\frac{S(u)-S_0(u)}{S(u)} \approx 5\%$. Taking into account a single recombination and healing event leads to increased accuracy (see e.g.[3] for a related approach). Using the above formulation, this amounts to considering the first term of the sum. Under a constant demographic model, the survival function is given by:

$$S_1(u \mid \gamma) = \frac{\gamma\left(3\gamma^2+4u^2+10\gamma u\right)}{(\gamma+2u)^2(3\gamma+2u)}, \tag{10}$$

which greatly reduces the relative error compared to the SMC approximation (e.g. $\sim 10\times$ lower using the previous example). This approach thus provides a good balance between accuracy

and computational cost, as it allows multiple expressions to be computed analytically if $\gamma(t)$ is approximated by a piece-wise constant function.

1.1.3 IBD model

We aim to model the number of IBD segments of particular lengths shared between pairs of individuals from a population. We denote the probability density function of the length of an IBD segment by $f_{seg}(l|\gamma(t))$, dropping the $\gamma(t)$ term for clarity. We first consider the length of an IBD segment spanning a given site x along the genome. The probability density function for the length of such a segment, $f_{site}(l)$, is related to $f_{seg}(l)$ through the following relation[2]:

$$\begin{aligned} f_{site}(l) &= \frac{l f_{seg}(l)}{\int_0^\infty l f_{seg}(l) dl} \\ &= \frac{l}{\mathbb{E}[L]} f_{seg}(l), \end{aligned} \tag{11}$$

where $\mathbb{E}[L]$ represents the expected length of a randomly selected IBD segment. The TMRCA of the two haplotypes at site x is distributed according to $f(t)$. Conditioned on a TMRCA t, the length of the IBD segments spanning x is the sum of the distances to the next change of ancestor on either side of the site. By allowing at most one healing event within the IBD segment as described above, the density takes the form:

$$\begin{aligned} f_{site}(l|t) &\approx (1 - p_h(t))^2 f_{erl}(l; 2t, 2) + 2p_h(t)(1 - p_h(t))^2 f_{erl}(l; 2t, 3) \\ &\approx (1 - 2p_h(t)) f_{erl}(l; 2t, 2) + 2p_h(t) f_{erl}(l; 2t, 3) + \mathcal{O}(p_h^2(t)), \end{aligned} \tag{12}$$

where the first term accounts for the case of no healing events and the second term allows for one recombination event. Marginalizing t, we obtain:

$$f_{seg}(l) = \frac{\mathbb{E}[L]}{l} \int_0^\infty f_{site}(l|t)\gamma(t)e^{-\Gamma(t)} dt. \tag{13}$$

For a constant demographic history, this becomes:

$$f_{seg}(l|\gamma) = \frac{12\gamma^2 \left(3\gamma^4 + 8l^4 + 52\gamma l^3 + 90\gamma^2 l^2 + 51\gamma^3 l\right)}{(\gamma + 2l)^4 (3\gamma + 2l)^3} \tag{14}$$

Neglecting the probability of healing leads to the SMC approximation for a constant demographic history:

$$f_{seg}^{SMC}(l|\gamma) = \frac{4\gamma^2}{(\gamma + 2l)^3}. \tag{15}$$

Conditioned on the total number of IBD segments N_s shared in a region, the expected count of IBD segments within a length bin delimited by u_i and u_{i+1} is $N_s \int_{u_i}^{u_{i+1}} f_{seg}(l)dl$. Furthermore, $\mathbb{E}[N_s] = \frac{L_c}{\mathbb{E}[L]}$, with L_c denoting the genomic length of the current region. Thus, the expected value of the number of segments within the ith bin Y_i is given by:

$$\mathbb{E}[Y_i] = L_c \int_{u_i}^{u_{i+1}} \int_{0}^{\infty} \frac{f_{site}(l|t)}{l} \gamma(t) e^{-\Gamma(t)} dt dl. \tag{16}$$

Note that we neglect issues due to finite size chromosomes, which we found to have a negligible effect. For a constant demographic history, this quantity becomes:

$$\mathbb{E}[Y_i] = L_c \left. \frac{2\gamma^2(8u^2 + 6u\gamma - 3\gamma^2)}{(2u+\gamma)^3(2u+3\gamma)^2} \right|_{u_{i+1}}^{u_i} \tag{17}$$

Supplementary Equation 16 provides the first moment of the distribution of Y_i. Note that the approximation introduced in Supplementary Equation. 10 allows to compute this expression analytically when the demographic model $\gamma(t)$ is a piece-wise constant function. Previous expressions derived under the full SMC', on the other hand, required the use of special functions or numerical integration[7].

Poisson distributions provide a natural way of describing "count data" such as Y_i. However, when using the Poisson model, we encountered bin-dependent overdispersion, particularly for smaller bins, where IBD segments originate from older coalescence events that likely involve multiple samples. We thus used a quasi-likelihood approach[17], adding a dispersion parameter ϕ_i:

$$f(y; \mu_i) = e^{\frac{y \log \mu_i - \mu_i}{\phi_i} - \log y!}, \tag{18}$$

where $\mu_i = \mathbb{E}[Y_i]$ and the Poisson mass function is recovered for $\phi_i = 1$. The dispersion parameters ϕ_i are set so that the variance of the deviance residuals is 1.

1.1.4 LD model

Rather than relying on the direct observation of IBD data, HapNe-LD leverages long-range correlations that are induced by shared segments, which may be detected using unphased data. To describe the LD model used by HapNe, we begin by noting that alleles found at high frequency in a sample are typically older than ancestors transmitting large IBD segments (also see Section 1.2.1 for calculations related to the age of IBD segments). This implies that high frequency mutations found on long IBD segments are also likely to be carried by the shared ancestor transmitting the segment. We restrict our analysis to sites with MAF > 0.25. Given one such high frequency site x, we assume that the haplotypes of two individuals i and j spanned by a large (> 0.5 cM) IBD segment satisfy

$$\mathbb{E}[X_i X_j | \text{IBD}] = \mathbb{E}[X^2], \tag{19}$$

and that the same haplotypes will be independent if not spanned by an IBD segment, i.e.

$$\mathbb{E}[X_i X_j | \neg\text{IBD}] = \mathbb{E}[X]^2. \tag{20}$$

The presence of IBD segments therefore leads to correlation in the observed genotypes, which HapNe-LD aims to leverage for the inference of effective population size variation. The input for HapNe-LD is a set of unphased genotypes $\tilde{G}_{x,i} = \tilde{X}_{i,1} + \tilde{X}_{1,2}$, where $i \in \{1, ..., s\}$ denote individuals in the panel, and $x \in \{1, ..., M\}$ denote sites. $\tilde{X}_{i,1}$ and $\tilde{X}_{i,2}$ represent the (hidden) haplotypes of sample i at site x, with $\tilde{X}_{i,1} \sim \text{Bernoulli}(p_x)$ where p_x is the population's allele frequency at site x. For simplicity, we consider standardized input data:

$$X_i = \frac{\tilde{X}_i - \hat{p}_x}{\sqrt{\hat{p}_x(1-\hat{p}_x)}}, G_{i,x} \equiv \frac{\tilde{G}_{i,x} - 2\hat{p}_x}{\sqrt{2\hat{p}_x(1-\hat{p}_x)}},$$

where $\hat{p}_x \equiv \frac{1}{s}\sum_{i=1}^{s} \tilde{X}_i$ is the estimator of the allele frequency at site x, which is assumed to remain constant in the recent past.

HapNe-LD leverages the LD between all pairs of sites (x, y), measured as:

$$\begin{aligned}\mathrm{LD}_{x,y} &= \frac{\sum\limits_{i=1}^{s}\sum\limits_{j=i+1}^{s} G_{i,x}G_{j,x}G_{i,y}G_{j,y}}{\binom{s}{2}} \\ &= \frac{\left(\sum\limits_{i=1}^{s} G_{i,x}G_{i,y}\right)^2 - \sum\limits_{i=1}^{s}\left(G_{i,x}G_{i,y}\right)^2}{s(s-1)},\end{aligned} \tag{21}$$

where s is the number of individuals in the dataset. Note that the correlations between all pairs present in the panel can be computed in $\mathcal{O}(s)$, whereas the detection of IBD segments requires $\mathcal{O}(s^2)$.

HapNe-LD aggregates these observations according to the genetic distance separating the sites x and y by computing:

$$R_i^2 = \frac{\sum\limits_{x,y} \mathrm{LD}_{x,y} I_{b_i}(\delta(x,y))}{\sum\limits_{x,y} I_{b_i}(\delta\,(x,y))}, \tag{22}$$

where $\delta(x, y)$ denotes the genetic distance in cM between the sites x and y, I_{b_i} is the indicator function for the interval b_i, and $b_i = (0.5 + 0.5i, 1 + 0.5i)$, $i \in \{0, 1, ...18\}$.

We now aim to relate these correlation statistics to the effective population size. The first moment of R_b^2 is given by:

$$\begin{aligned}\mathbb{E}[R_b^2] &= \mathbb{E}[G_{i,x}G_{j,x}G_{i,y}G_{j,y}] \\ &= \sum_{\alpha,\beta,\gamma,\delta\in\{1,2\}} \frac{1}{4}\mathbb{E}[X_{i,\alpha}X_{j,\beta}Y_{i,\gamma}Y_{j,\delta}].\end{aligned} \tag{23}$$

We can group the 16 terms of the sum into different categories, according to the number of distinct haplotypes involved in each of these terms. In particular, the 4 terms where $\alpha = \gamma$ and $\beta = \delta$ involve two distinct haplotypes, i.e. haplotype α for individual i and β for individual j. For these 4 terms, we can use supplementary equations 10, 19, and 20 to write:

$$\begin{aligned}\mathbb{E}[X_{i,1}X_{j,1}Y_{i,1}Y_{j,1}] &= \mathbb{E}[X_{i,1}X_{j,1}Y_{i,1}Y_{j,1}|\mathrm{IBD}(x,y)]S_1(u) + \mathbb{E}[X_{i,1}X_{j,1}Y_{i,1}Y_{j,1}|\neg\mathrm{IBD}(x,y)](1 - S_1(u)) \\ &= (\mathbb{E}[X^2Y^2] - \mathbb{E}[XY]^2)S_1(u) + \mathbb{E}[XY]^2 \\ &= S_1(u),\end{aligned} \tag{24}$$

where u denotes the distance between the two sites x and y. Note that we neglect issues due to finite sample sizes and population structure, which are addressed later. With this assumption, we have $\mathbb{E}[X^2Y^2] = \mathbb{E}[X^2]\mathbb{E}[Y^2] = 1$ and $\mathbb{E}[XY] = 0$.

The 12 other terms of the sum of Supplementary Equation 23 involve either 3 or 4 haplotypes. For example, a term with $\alpha \neq \gamma$ and $\beta = \delta$ involves both haplotypes for individual i and haplotype β for individual j. In these cases, correlations induced by IBD require at least two pairs of haplotypes to be shared IBD, leading to $\mathcal{O}(S_1^2(u))$ contributions, which we neglect.

Together, these expressions enable obtaining the first moment of R_b^2. If bin b is delimited by u_i and u_j, we have:

$$\mathbb{E}[R_b^2] = \mu_b = \frac{1}{u_j - u_i} \int_{u_i}^{u_j} S_1(u) du. \tag{25}$$

To complete the model, we assume that

$$R_b^2 \sim \mathcal{N}(\mu_b, \sigma_b^2) \tag{26}$$

and estimate σ_b^2 using $R_{b,r}^2$ estimates obtained across chromosome arms.

1.1.5 Correcting for finite sample size

Working with finite sample sizes induces correlations in the data which, if not accounted for, lead to bias in the inferred effective population size. These correlations arise as a result of the use of an empirical allele frequency $\hat{p}_x$ instead of the unknown p_x. As a first step to debias the estimator of R^2, we consider the ratio of the expected values as an approximation to the expected value of the ratio, which has been shown to be a good approximation for common alleles[18]:

$$\mathbb{E}[X_i X_j] \approx \frac{\mathbb{E}[(\tilde{X}_i - \hat{p}_x)(\tilde{X}_j - \hat{p}_x)]}{\mathbb{E}[\hat{p}_x(1 - \hat{p}_x)]} \tag{27}$$

Let s_x denote the number of haplotypes observed at site x, i.e. twice the number of individuals in modern datasets or the number of individuals in pseudo-haploid aDNA data. We can rewrite

the numerator as:

$$
\begin{aligned}
&\mathbb{E}\left[\left(\tilde{X}_i - \frac{1}{s_x}\sum_{k=1}^{s_x}\tilde{X}_k\right)\left(\tilde{X}_j - \frac{1}{s_x}\sum_{k=1}^{s_x}\tilde{X}_k\right)\right] \\
&= \mathbb{E}[\tilde{X}_i\tilde{X}_j] - \frac{2}{s_x}\mathbb{E}[\tilde{X}_i^2] - \frac{2}{s_x}\mathbb{E}[\tilde{X}_i\sum_{k\neq i}\tilde{X}_k] + \mathbb{E}[(\sum_{k=1}^{s_x}\tilde{X}_k)^2] \\
&= \frac{-p_x(1-p_x)}{s_x}
\end{aligned} \tag{28}
$$

Similarly, the denominator is given by:

$$\mathbb{E}[\hat{p}_x(1-\hat{p}_x)] = \frac{s_x - 1}{s_x}p_x(1-p_x) \tag{29}$$

It follows that:

$$\mathbb{E}[X_iX_j] = \frac{-1}{s_x - 1} \neq 0 \tag{30}$$

When working with low coverage data, s_x becomes a random quantity, S_x, as some individuals are not genotyped at site x. Because computing LD between x and y requires that at least two individuals are sequenced at both sites, S_x and S_y are not independent for the (x, y) pairs considered when computing LD. We therefore average realizations of $\frac{1}{(S_x-1)(S_y-1)}$ over pairs of sites (x, y) to compute an estimate $\hat{\beta}$ for the following quantity in Supplementary Equation 24:

$$\mathbb{E}[X_iX_jY_iY_j|\neg\text{IBD}] = \mathbb{E}[\frac{1}{(S_x-1)(S_y-1)}] \equiv \beta, \tag{31}$$

which is also relevant for the detection of population structure, as discussed later. We use the same pairs (x, y) to similarly obtain an estimate $\hat{\alpha}$ for the quantity

$$\mathbb{E}[X^2Y^2|\text{IBD}] \approx \mathbb{E}[\frac{(S_x^2 - S_x + 2)(S_y^2 - S_y + 2)}{(S_x^2 - 3S_x + 2)(S_y^2 - 3S_y + 2)}] \equiv \alpha, \tag{32}$$

and use these terms to obtain a corrected estimate for R_b^2

$$\hat{R_b^2} = (\hat{\alpha} - \hat{\beta})S_1(u;\gamma(t)) + 4\hat{\beta}. \tag{33}$$

Note that the factor 4 is due to the $\mathcal{O}(S_1(u)^2)$ terms in Supplementary Equation 23 that also

cause finite-sample size correlations.

1.1.6 Correcting for time heterogeneity

Ancient DNA samples in a data set often originate from different time points. Due to the uncertainty in obtaining precise time estimates, their origins are often reported as a time range. Time heterogeneity across the set of analyzed samples causes a reduction in LD, due to the effects of recombination on the underlying haplotypes. If not modeled, this leads to an upwards bias in the estimated effective population size. HapNe-LD implements a correction to prevent these biases using the reported sample ages, which are obtained via radio-carbon dating or using the archeological context.

Consider two individuals i and j sampled at times T_i and T_j. Assume, without loss of generality, that $T_i > T_j$ and define $\Delta T \equiv T_i - T_j > 0$. Following the lineage of individual j at a site x, we denote by k the ancestor living at generation T_i. The LD between individuals i and k, both of them living at generation T_i, can be computed using Supplementary Equation 7 by replacing $\gamma(t)$ with $\gamma_o(t) = \gamma(t + T_i)$. The LD between individuals i and j is obtained by multiplying the LD between individuals i and k by the probability that the haplotype is not broken by a recombination event when transmitted from k to j, which decays exponentially with rate ΔT. Under the SMC approximation, this probability is given by $e^{-\Delta T u}$. In practice, T_i and T_j are not known exactly but provided as a range. If the density functions of T_i and T_j are available, both times can be marginalized in the above calculations of LD. HapNe supports used-provided time intervals for each sample and assumes that the true time is uniformly distributed within these intervals.

1.1.7 Population Structure

Population structure causes correlation due to differences in allele frequencies across diverged populations. This correlation may lead to biases in the inferred demographic models. We use Supplementary Equation 31 to detect the presence of population structure and partially correct for it. For each pair of distinct chromosomes i and j, we compute the average difference between both sides of Supplementary Equation 31 and use a two-sided t-test to verify that they do not significantly deviate from 0. To mitigate the effects of population structure, we estimate $\mathbb{E}[X_i X_j Y_i Y_j | \neg \text{IBD}]$ by averaging realizations of $X_i X_j Y_i Y_j$ for loci located on different chromosomes, and used this value as an estimate of β in Supplementary Equation 33. Note that,

because all pairs of chromosomes are used to compute the t-test, the samples are not strictly independent, making this approach slightly conservative. An alternative approach consists in only considering disjunct pairs of chromosomes, which however leads to higher variance in the estimates for β.

As shown in Supplementary Figure 9, this approach, implemented in HapNe-LD, leads to unbiased results in our simulations, whereas other methods that do not consider LD induced by structure lead to a downward bias in the estimated effective population size. Note, however, that this approach does not correct for admixture LD, which decays with the genetic distance (see Supplementary Figure 10).

1.1.8 Effective population size in multi-population models

We used the backward-in-time Markov chain introduced in[19] to convert coalescence rates for the multi-population models into effective sizes for an equivalent single-population model. In particular, given a demographic model involving multiple populations, we used a Markov chain to compute the probability that two lineages coalesce at generation t, conditioned on not having coalesced up to generation $t-1$, and took the inverse of this probability to be the effective population size for an equivalent single-population model.

1.1.9 Link between F_{st} and t_{split} in the simulations with admixture

In the Methods section, the simulated models involving admixture events are described in terms of two parameters, t_{split}, and t_{adm}. t_{split} corresponds to the time, in generations, at which an ancestral population of size N_e splits into two isolated populations A and B of constant size $N_e/2$, while t_{adm} is the time at which a new population is created from these two isolated populations. When discussing these simulations in the Results section, we report the value of F_{st} between populations A and B in these simulated models, which is linked to t_{split} by the expression:

$$F_{st} = 1 - \frac{2\left(1 + e^{-2\Delta_t\gamma}\right)}{\left(1 + e^{-2\Delta_t\gamma}\right) + 2\gamma\Delta_t + 2} \tag{34}$$

where $\Delta_t \equiv t_{split} - t_{adm} > 0$ is the isolation time, in generations. This follows from the relationship between F_{st} and the ratio between the expected coalescent time for two haplotypes sampled from the same population T_w and the expected coalescent time for a pair of haplotypes

sampled in the combined populations T_{all}[20]:

$$F_{st} = 1 - \frac{T_w}{T_{all}}, \tag{35}$$

Defining the coalescent rate in the ancestral population as $\gamma \equiv \frac{1}{N_e}$, the expected coalescent time for lineages sampled from the same population is given by:

$$\begin{aligned} T_w &= \int_0^{\Delta_t} t2\gamma e^{-2\gamma t} dt + \int_{\Delta_T}^{\infty} t\gamma e^{-2\Delta_t\gamma-(t-\Delta_t)\gamma} dt \\ &= \frac{1+e^{-2\Delta_t\gamma}}{2\gamma} \end{aligned} \tag{36}$$

Because both populations equally contribute to the admixture event, we have $T_{all} = 0.5\,(T_w + T_{across})$, where T_{across} is the expected coalescent time for lineages sampled from different populations. Since there is no migration between populations A and B and the ancestral population has size N_e, the expected coalescent time for lineages sampled across populations is:

$$T_{across} = \Delta_t + N_e, \tag{37}$$

leading to Supplementary Equation 34.

1.2 Additional details on the inference procedure

We provide additional details on the use of quantiles of the IBD segment age distribution to discretize the time intervals and on the regularized loss function minimized by HapNe to infer $N_e(t)$.

1.2.1 Parameterization of $N_e(t)$

HapNe aims to infer the demographic model given by $N_e(t)$. We parameterize this function by assuming it to be piece-wise exponential, with parameters described by a vector, θ. More in detail, we divide the time axis into M consecutive intervals and for each interval i assume that $N_e(t)$ varies according to a constant exponential rate λ_i. We set $\lambda_M = 0$, implying that the population size remains constant from the last predicted time to infinity. $N_e(t)$ is thus fully determined by a set of M values $\theta = \{N_0, \{\lambda_i\}_{i=1\ldots M-1}\}$. This parametrization is motivated by the fact that changes from one generation to the other are proportional to the size of the

population, and has been shown to help addressing issues with identifiability in a related context[21,22].

Time intervals are automatically selected so that each of them contains the same expected number of IBD segments (as also done in e.g.[23]). Let $f_{age}(t|l > u_{min})$ denote the probability density function of the age of IBD segments whose length satisfies $l > u_{min}$. We define time intervals so that they coincide with quantiles of this density, which we compute using

$$f_{age}(t|l > u_{min}) = \frac{\int\limits_{u_{min}}^{\infty} f_{age}(t|l)f_{seg}(l)dl}{1 - F_{seg}(u_{min})}, \tag{38}$$

where $f_{seg}(u)$ in defined in Supplementary Equation 13 and $F_{seg}(u) = \int\limits_{0}^{u} f_{seg}(l)dl$. To derive $f_{age}(t|l)$, we note that it represents the TMRCA of a randomly selected site spanned by an IBD segment of length l. Using Bayes' rule and the SMC approximation,

$$\begin{aligned} f_{age}(t|l) &= \frac{f_{site}(l|t)f(t)}{f_{site}(l)} \\ &= \frac{(2t)^2 l e^{-2tl}\gamma(t)e^{-\Gamma(t)}}{\int\limits_{0}^{\infty}(2t)^2 l e^{-2tl}\gamma(t)e^{-\Gamma(t)}dt}. \end{aligned} \tag{39}$$

For a constant coalescent rate γ, this becomes

$$\begin{aligned} f_{age}(t|l) &= \frac{1}{2}t^2(2l+\gamma)^3 e^{-(\gamma+2l)t} \\ f_{age}(t|l > u) &= t(2u+\gamma)^2 e^{-(\gamma+2u)t}, \end{aligned} \tag{40}$$

i.e. an Erlang-3 and Erlang-2 distribution, respectively (also see[6,9]). Because time intervals depend on $N_e(t)$, HapNe iteratively tunes them at each iteration using the current population size estimates.

Note that a slightly more accurate closed-form solution under a constant population size can be obtained by allowing a single recombination event to heal, replacing f_{site} in Eq. 39 with the expression of Eq. 12, leading to:

$$f_{age}(t|l) = \frac{t(\gamma+2l)^4(3\gamma+2l)^3 e^{-2lt-3\gamma t}\left(e^{2\gamma t}(lt(2\gamma t-1)+1)+lt-1\right)}{8\gamma\left(3\gamma^4+8l^4+52\gamma l^3+90\gamma^2 l^2+51\gamma^3 l\right)} \tag{41}$$

1.2.2 Loss function

We aim to find the best set of parameters θ based on correlated observations $Y = \{y_{r,b}\}$, where $y_{r,b}$ represents LD or IBD summary statistics computed for the bth bin of the rth independent genomic region. Due to the presence of correlations in the data, rather than using standard likelihood calculations we work with the approximated power likelihood

$$p(Y|\theta) = \prod_{r,b} f_b(y_{r,b};\theta)^c, \tag{42}$$

where $0 \leq c \leq 1$ is a hyperparameter and f_b is the probability mass or density function derived in supplementary equations 18 and 26. Minimizing Eq. 42 for θ is an ill-defined problem, for which small changes in the input data might lead to significant changes in the inferred parameter $\hat{\theta}$ (also see e.g.[4]). To improve convergence and restrict the parameter space we thus impose the following prior on the $\{\lambda\}$ coefficients of the piece-wise exponential function $N_e(t)$:

$$p_{N_e}(\{\lambda_i\}) \propto e^{-\frac{\sum_{i=1}^{M-1} \Delta t_i \sqrt{(\lambda_i^2+1)}}{2\sigma^2}}, \tag{43}$$

where Δt_i denotes the length of the ith time interval and λ_i the growth rate in the same interval, and σ^2 is a hyperparameter, which we discuss in more detail below.

The terms in the numerator of Supplementary Equation 43 may be rewritten as

$$\Delta t_i \sqrt{(\lambda_i^2 + 1)} = \sqrt{|\log \frac{N_{i+1}}{N_i}|^2 + \Delta t_i^2}, \tag{44}$$

highlighting the relationship between this quantity and the arc length of $\log N_e(t)$ between $t = 0$ and t_M.

Hence, this prior favors simple demographic models, such as constant population size or a single exponential growth. We found this choice of prior to be more effective than other approaches, such as using an L_1 or L_2 penalty on the coefficients λ.

Combining these expressions leads to the following posterior:

$$\log p(\theta|Y) \approx c \sum_{r,b} \log f_b(y_{r,b};\theta) + \sum_{i=1}^{M} \log p_{N_e}(\{\lambda_i; 0, \sigma^2\}) + Z, \tag{45}$$

where Z is a normalizing constant.

We aim to find the MAP of θ:

$$\begin{aligned}
\hat{\theta} &= \underset{\theta}{\text{argmax}}\ c \sum_{r,b} \log f_b(y_{r,b};\theta) - \sum_{i=1}^{M} \frac{\sqrt{\lambda_i^2 + 1}\Delta t_i}{2\sigma^2} \\
&= c \left[\underset{\theta}{\text{argmax}} \sum_{r,b} \log f_b(y_{r,b};\theta) - \sum_{i=1}^{M} \frac{\sqrt{\lambda_i^2 + 1}\Delta t_i}{2c\sigma^2} \right] \\
&= \underset{\theta}{\text{argmax}} \sum_{r,b} \log f_b(y_{r,b};\theta) - \sum_{i=1}^{M} \frac{\sqrt{\lambda_i^2 + 1}\Delta t_i}{2c\sigma^2}
\end{aligned} \tag{46}$$

This requires tuning a single hyperparameter $\kappa = c\sigma^2$, using the approach described in the next section.

1.2.3 Numerical optimization

We used SciPy's implementation of the L-BFGS-B optimiser[24] to minimize Supplementary Equation 46. Each minimization step is run 5 times using different starting points. The solution yielding the smallest loss is kept.

1.3 Model selection

HapNe performs a grid-search over different values of the hyperparameter κ, ranging from a strong regularization $\kappa_0 = 10^{-5}$ to an almost unregularized model with parameter $\kappa_{max} = 100$. For each of these parameters, HapNe infers the MAP $\hat{\theta}(\kappa)$ by optimizing Supplementary Equation 46, as well as the associated pseudo-likelihood $l_\kappa = \sum_{r,b} \log f_b(y_{r,b}; \hat{\theta}(\kappa))$. HapNe then computes the "pseudo-deviance" $D(\kappa) = 2(\log l_{\kappa_{max}} - \log l_\kappa)$. The smallest value of κ satisfying $D(\kappa) < \tau$ is selected as the best hyperparameter. Since the parameter c handling correlations between bins is neglected when computing the "pseudo-deviance", we cannot use asymptotic theories about the distribution of D to fix the value of τ in a principled way. Instead, we fixed the thresholds τ for both HapNe-LD and HapNe-IBD by training them using three sets of simulations that used different demographic models than the ones presented in this work.

1.4 Supplementary Figures

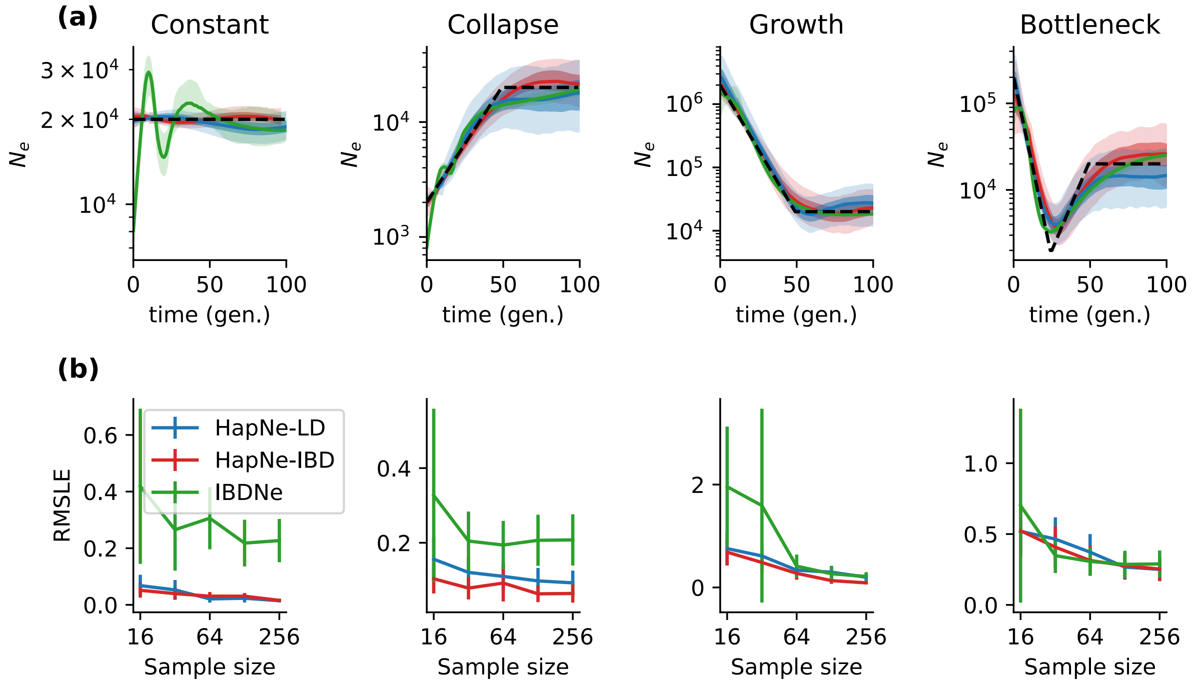

Supplementary Figure 1. **Accuracy of HapNe-IBD and IBDNe using ground truth IBD sharing information, and HapNe-LD using inferred LD.** (a) Simulated demographic models (dotted black lines), predictions based on ground truth IBD sharing for both HapNe-IBD (red) and IBDNe (green), and HapNe-LD results based on simulated SNP-array data (blue). (b) Error as a function of sample size for corresponding demographic models in (a), measured as the RMSLE over the first 50 generations (see Methods). HapNe-IBD and IBDNe were run using ground truth IBD sharing information. Error bars correspond to $1.96 \times SE$ computed using 10 independent simulations.

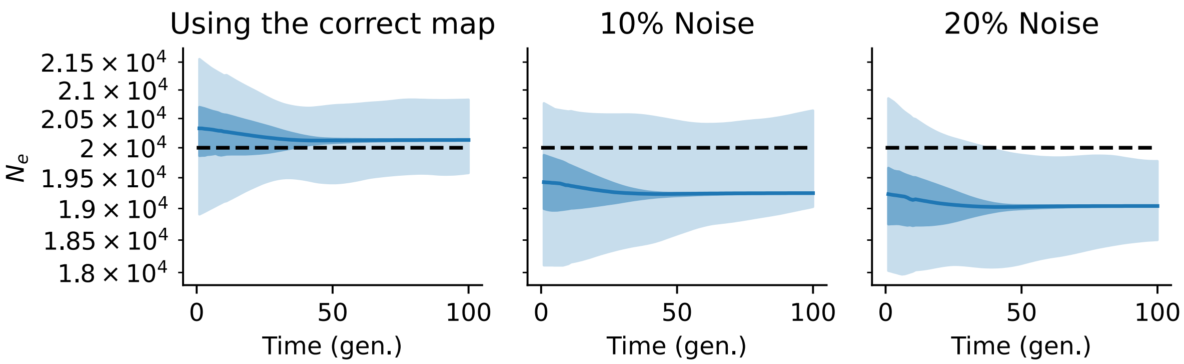

Supplementary Figure 2. **Effect of a misspecified genetic map on the inferred population sizes.** The left panel shows results obtained based on a genome-wide analysis of 256 diploid samples simulated using a GrCH37 genetic map. The central panel illustrates results obtained using the same simulated files, but after adding noise to the recombination map provided in input to HapNe. For each recombination rate value in the map, we added noise drawn from a normal distribution with 0 mean and a standard deviation of 10% (central panel) or 20% (right panel) of the true value.

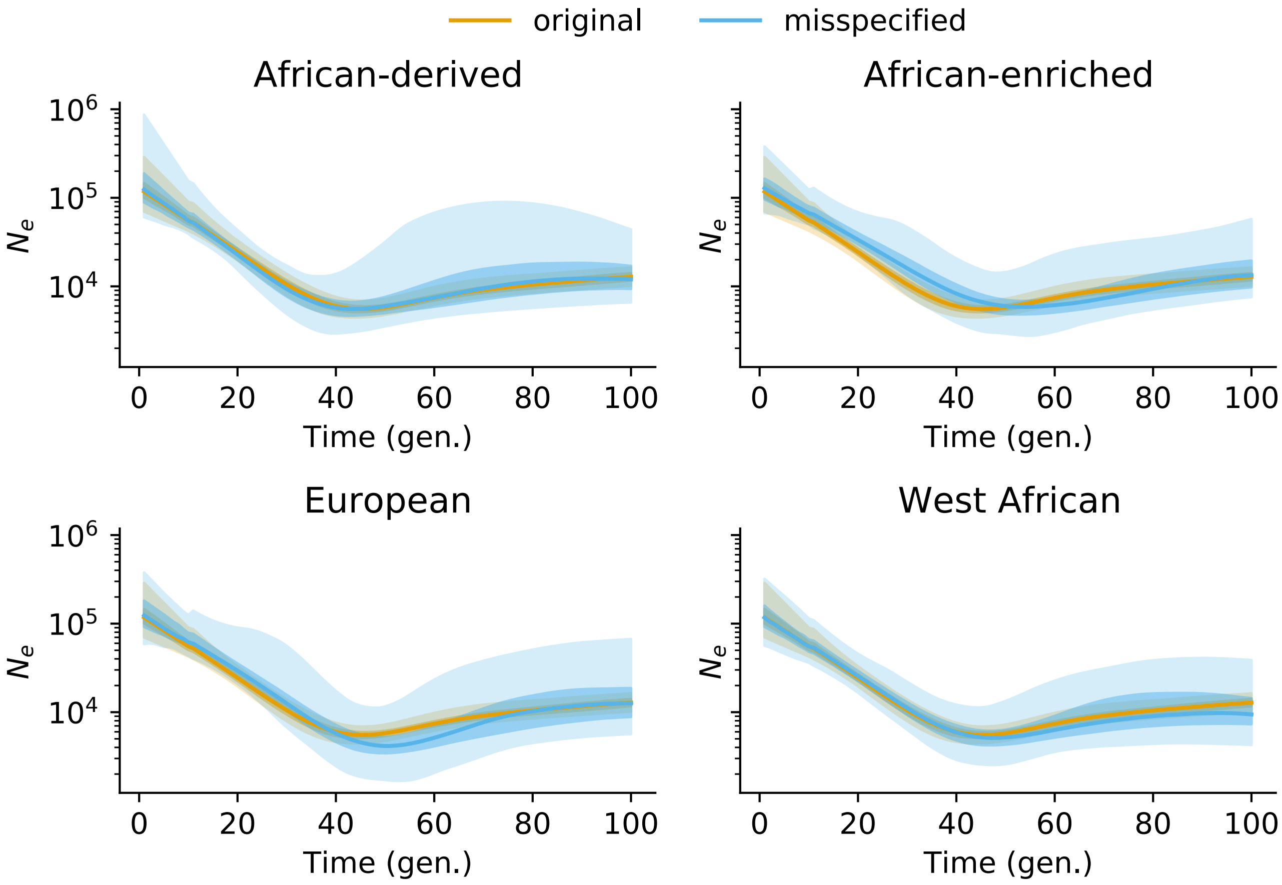

Supplementary Figure 3. **Inference of Finnish effective population size in the 1000 Genomes Project under different genetic maps**. In each panel, the result reported in Figure 4 of the main text (original) is displayed together with the inferred demography when another genetic map is used (misspecified). Genetic maps are described in Hinch et al[25].

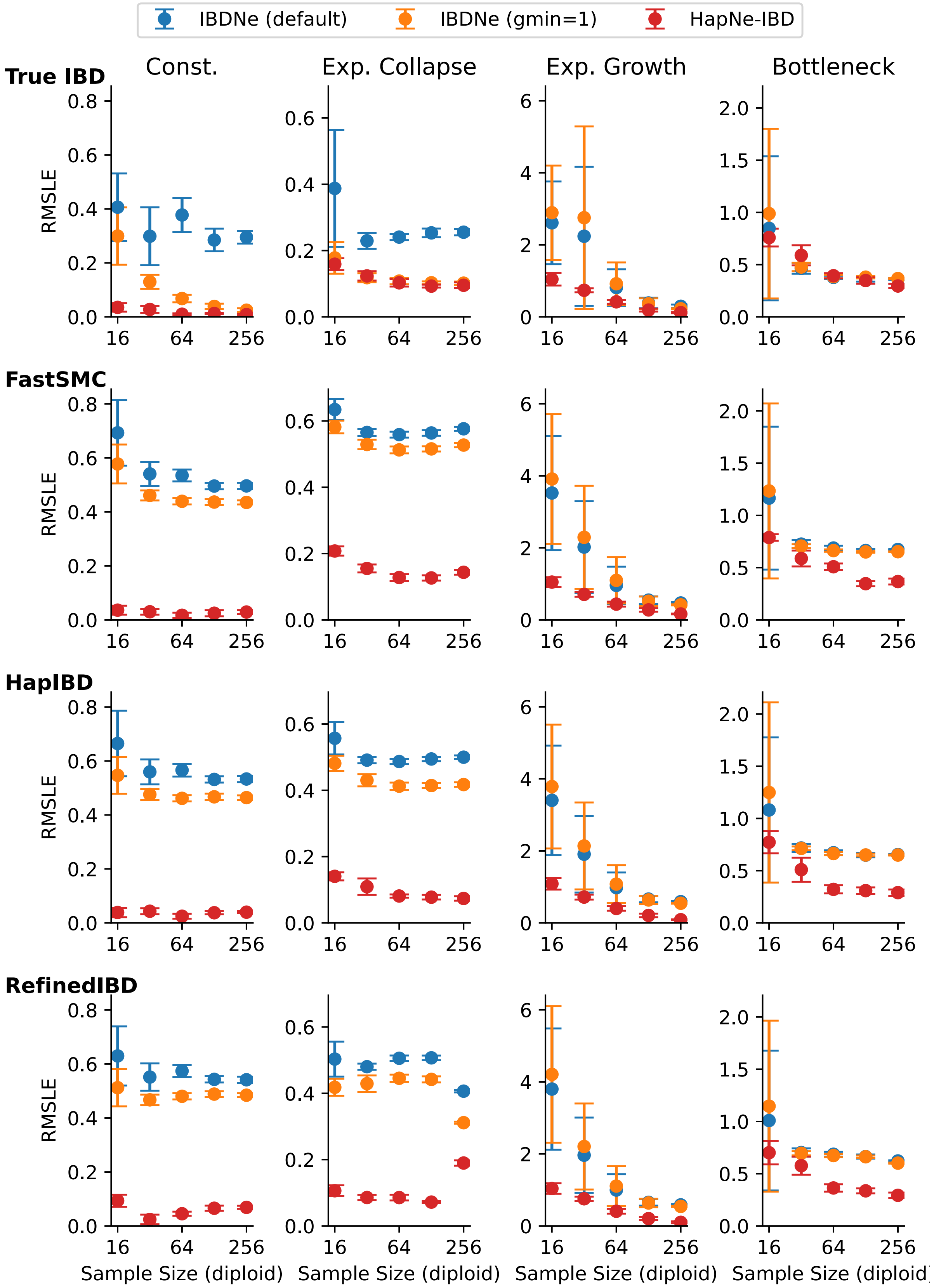

Supplementary Figure 4. **Impact of IBD detection on the accuracy of IBDNe and HapNe-IBD.** RMSLE as a function of sample size for IBDNe and HapNe-IBD. True IBD refers to the IBD segments obtained from the ARGON simulator. The IBD segments obtained using HapIBD and RefinedIBD were post-processed using the procedure described in the Methods section. We ran IBDNe with its default parameters (default) and with a set of parameters optimized for simulated data (gmin=1, see Methods).

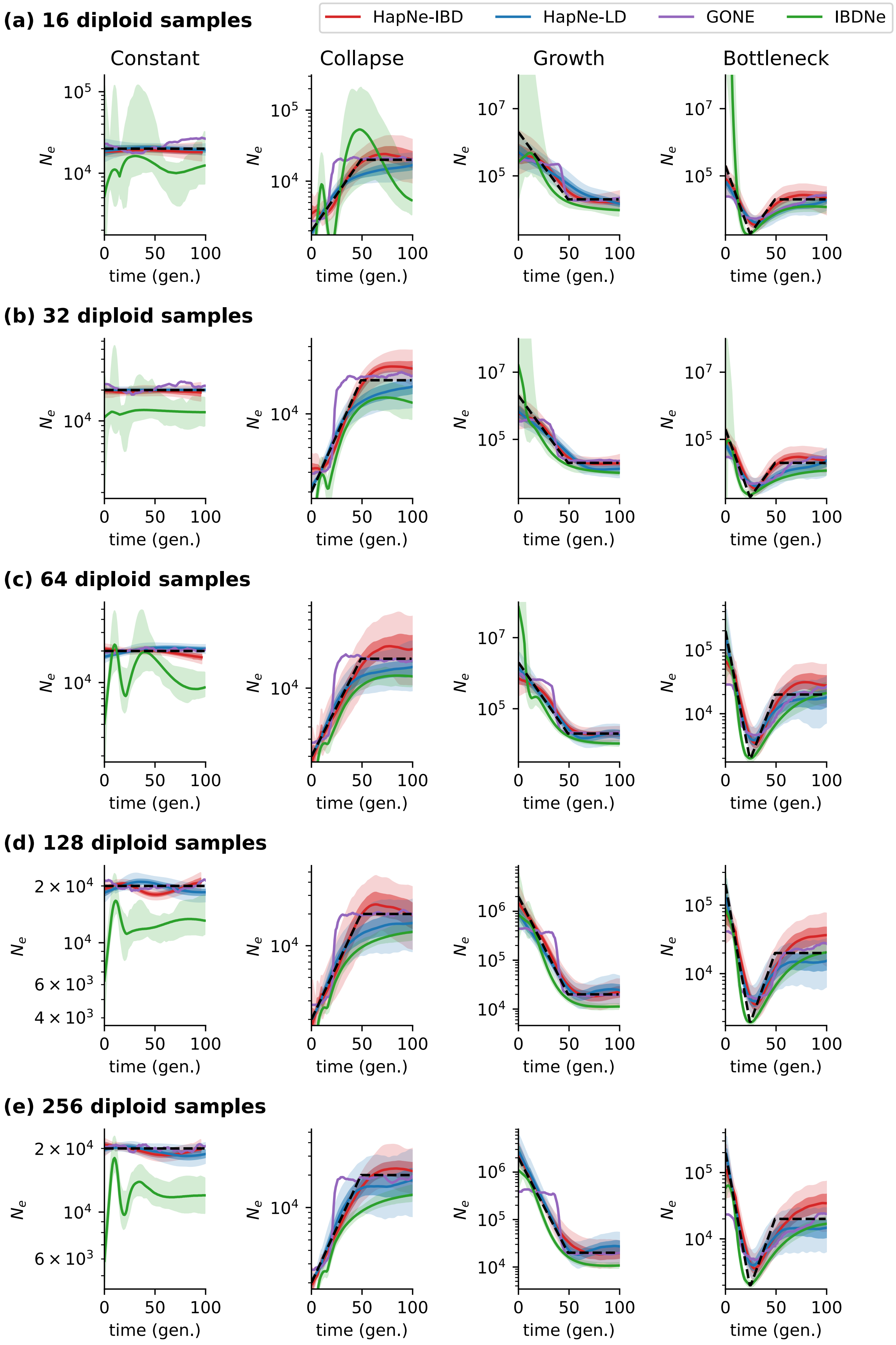

Supplementary Figure 5. **Effect of sample size variation (panels a-e) across several demographic models (columns).** HapNe-IBD was run using IBD segments detected by FastSMC and IBDNe using segments detected by HapIBD. LD methods were run using their standard pipeline. The y-axis is truncated for readability in simulations that resulted in very large values.

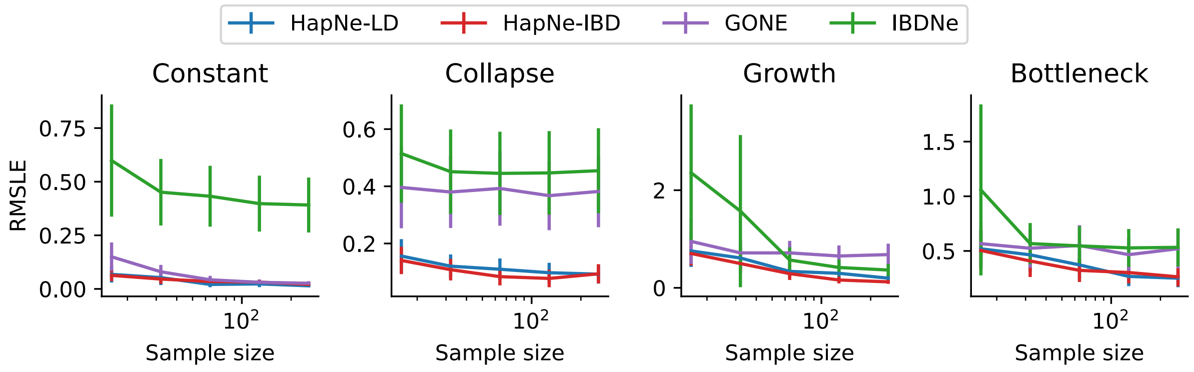

Supplementary Figure 6. **Inference accuracy as a function of sample size.** Accuracy was measured using RMSLE over the first 50 generations for each simulated demographic history and sample size (see Methods). IBD segments for HapNe-IBD and IBDNe were computed using FastSMC and HapIBD, respectively. Error bars correspond to 1.96 × SE computed using 10 independent simulations.

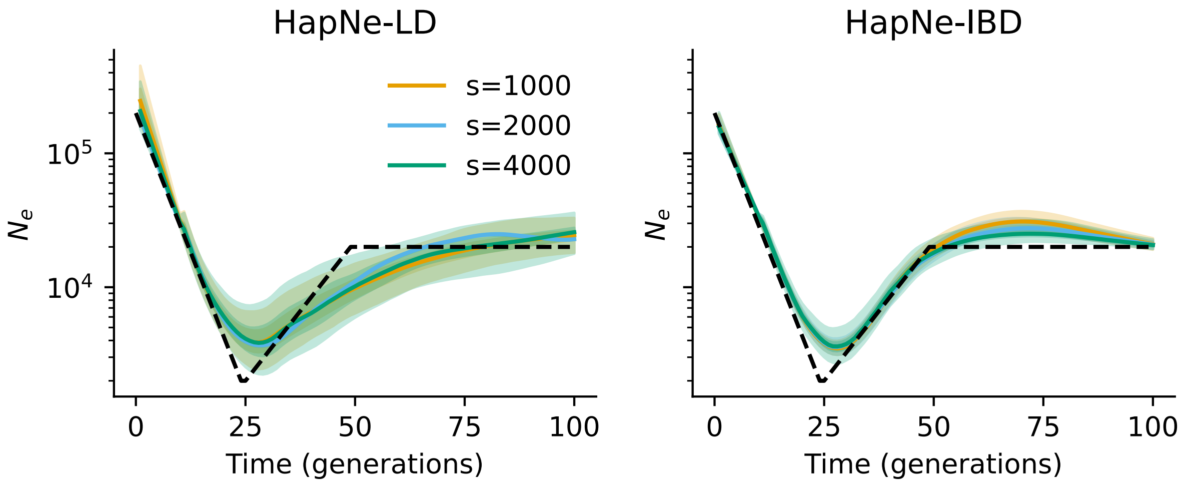

Supplementary Figure 7. **Inference results at larger sample sizes.** Results on HapNe-LD (left) and HapNe-IBD (right) for simulated data sets up to $s = 4,000$ individuals. IBD segments were detected using HapIBD, using perfectly phased synthetic data.

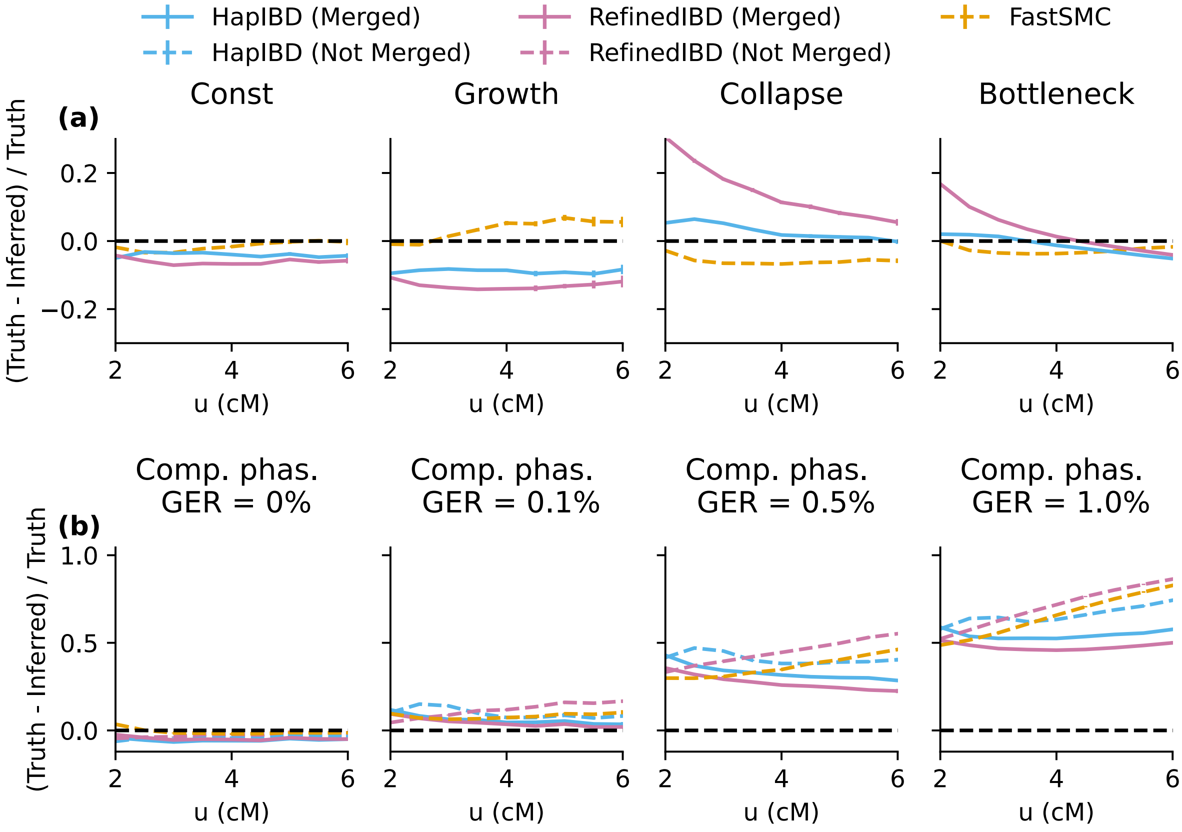

Supplementary Figure 8. **Evaluation of IBD detection accuracy in simulated data.** Evaluation of IBD detection accuracy in simulated data. (a) The relative error between the true and inferred number of IBD segments is shown for different IBD segment lengths, using three IBD detection methods, FastSMC, HapIBD, and RefinedIBD (see Methods). The results were obtained using 256 simulated phased diploid genotypes for different demographic histories. (b) The same analysis was performed for the constant demographic history, using computationally phased data based on 256 diploid individuals with increasing levels of genotyping error. Solid lines, labeled as Merged, correspond to post-processed outputs for which segments predicted to be broken due to phasing or genotyping error are merged (see Methods). Dashed lines correspond to the raw output of the IBD detection methods.

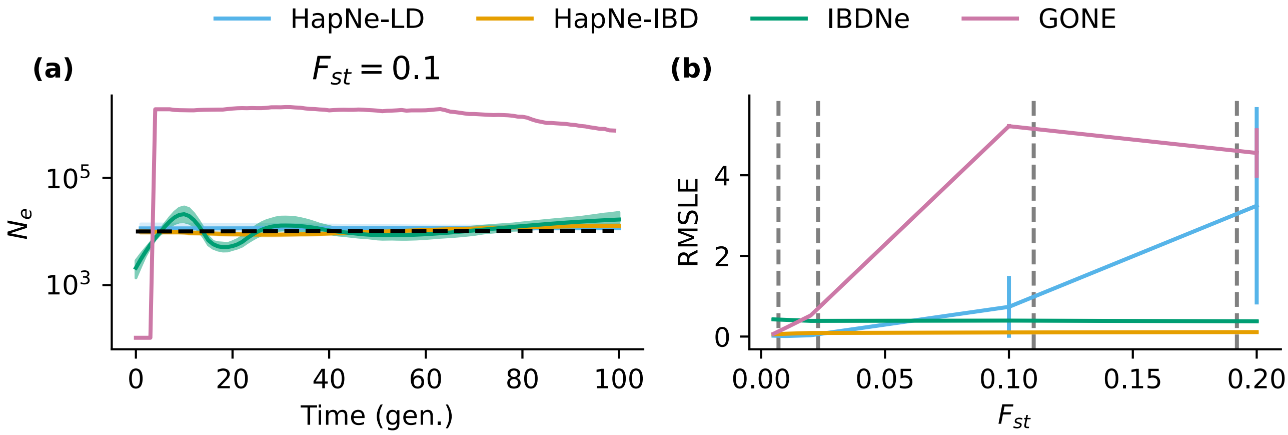

Supplementary Figure 9. **Impact of population structure on inference results.** (a) The output of HapNe-LD, HapNe-IBD, GONE, and IBDNe based on 100 samples evenly split between two separated populations with an F_{st} of 0.1. (b) Root mean square log-error (RMSLE) for increasing values of F_{st} between the two populations. From left to right, the vertical lines correspond to the estimated value of F_{st} between representative pairs of human populations from the HapMap Project and other European cohorts[26]: CHB and JPT (0.007), Finland Kuusamo and Southern Italy (0.023), CEU and CHB (0.11), and JPT and YRI (0.192). HapNe-IBD and IBDNe were run on IBD segments detected using HapIBD (see Methods). Dashed black lines correspond to the inverse of the coalescent rate (see Supplementary Note, Section 1.1.8).

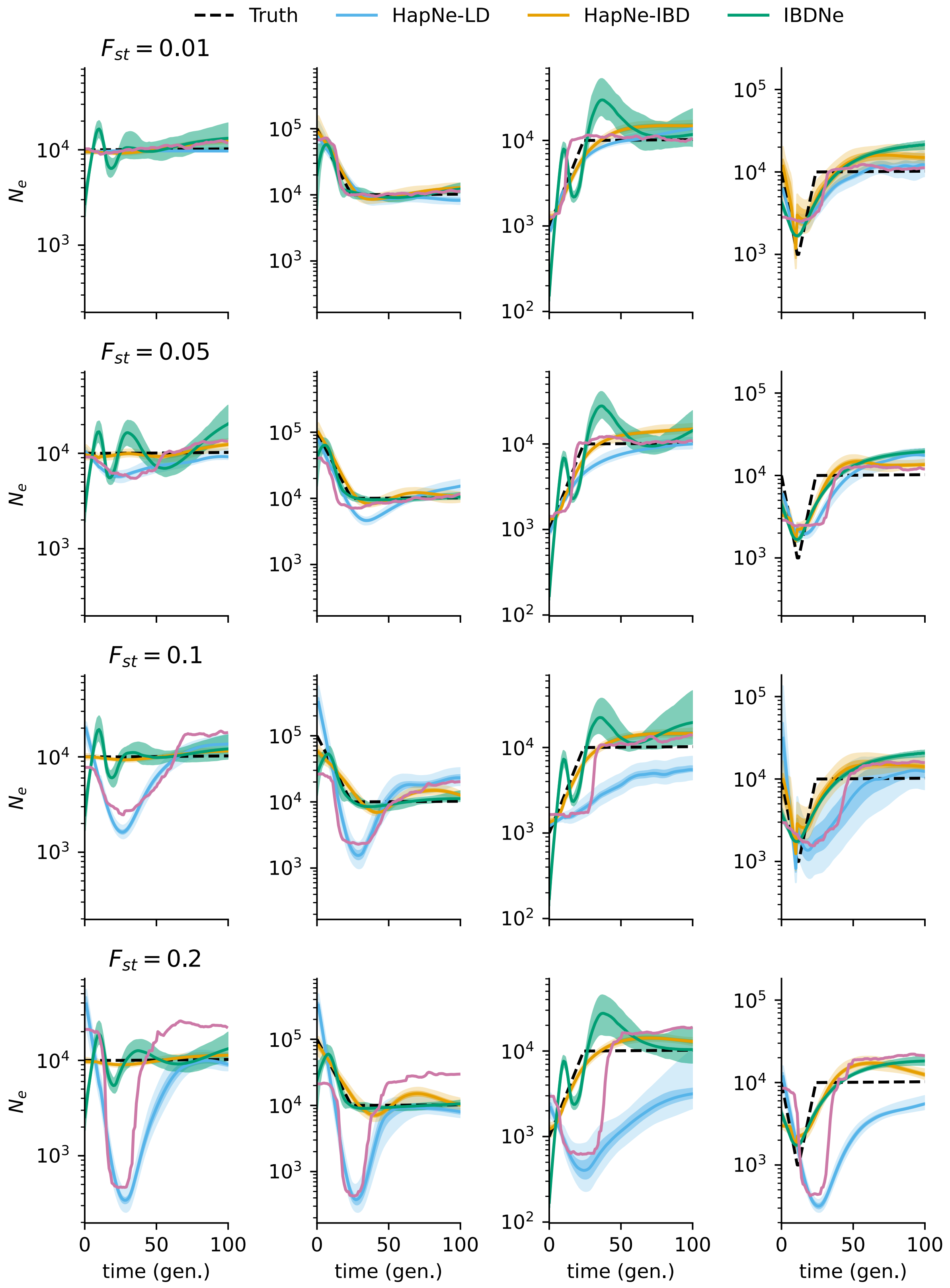

Supplementary Figure 10. **Impact of admixture on inference results.** The plots show the output of HapNe-LD, HapNe-IBD, GONE, and IBDNe based on 100 samples from a population originating from an admixture event 25 generations before present (see demographic model in Figure 2d of the main text). The F_{st} between the two ancestral populations involved in admixture is shown for each row. HapNe-IBD and IBDNe were run on IBD segments detected using HapIBD (see Methods). Dashed black lines correspond to the inverse of the coalescent rate (see Supplementary Note, Section 1.1.8).

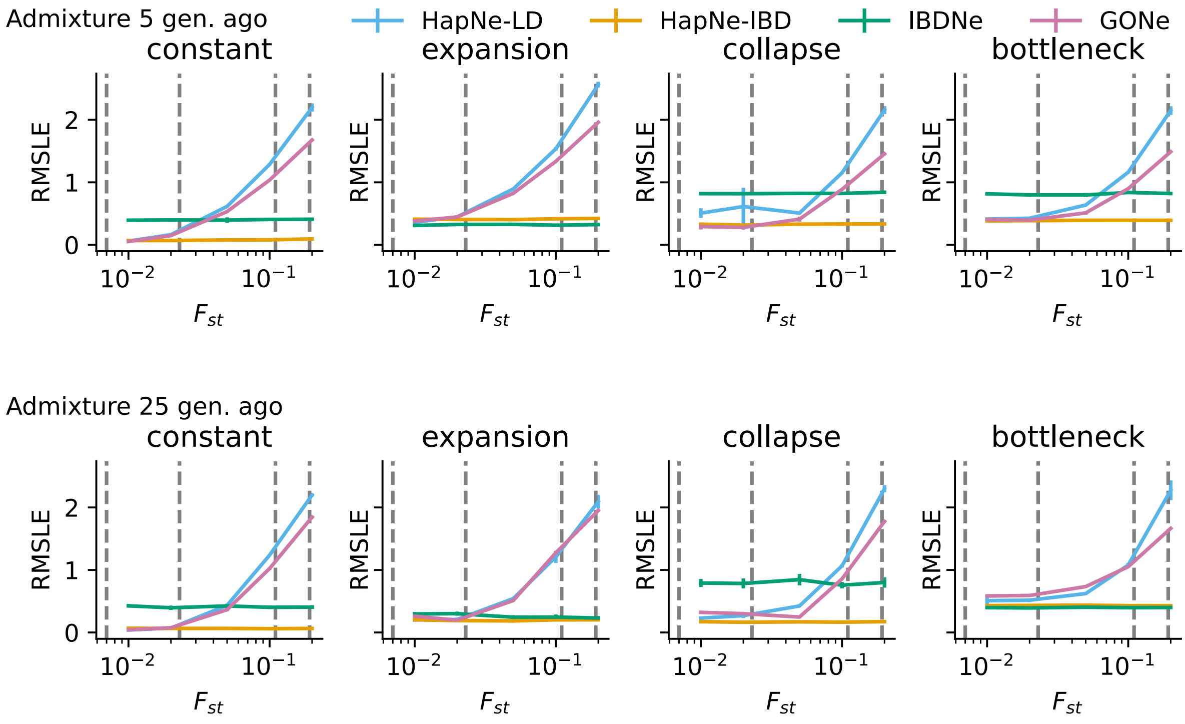

Supplementary Figure 11. **Effect of F_{st} between ancestral populations on HapNe inference results in scenarios involving admixture** Root mean square log-error (RMSLE) for a population originating from an admixture event (see demographic model in Figure 2d of the main text) 5 generations before present (first row) and 25 generations before present (second row). The RMSLE is plotted against the value of the F_{st} between the ancestral populations at the time of the admixture. From left to right, the vertical lines correspond to the estimated value of F_{st} between representative pairs of human populations from the HapMap Project and other European cohorts[26]: CHB and JPT (0.007), Finland Kuusamo and Southern Italy (0.023), CEU and CHB (0.11), and JPT and YRI (0.192).

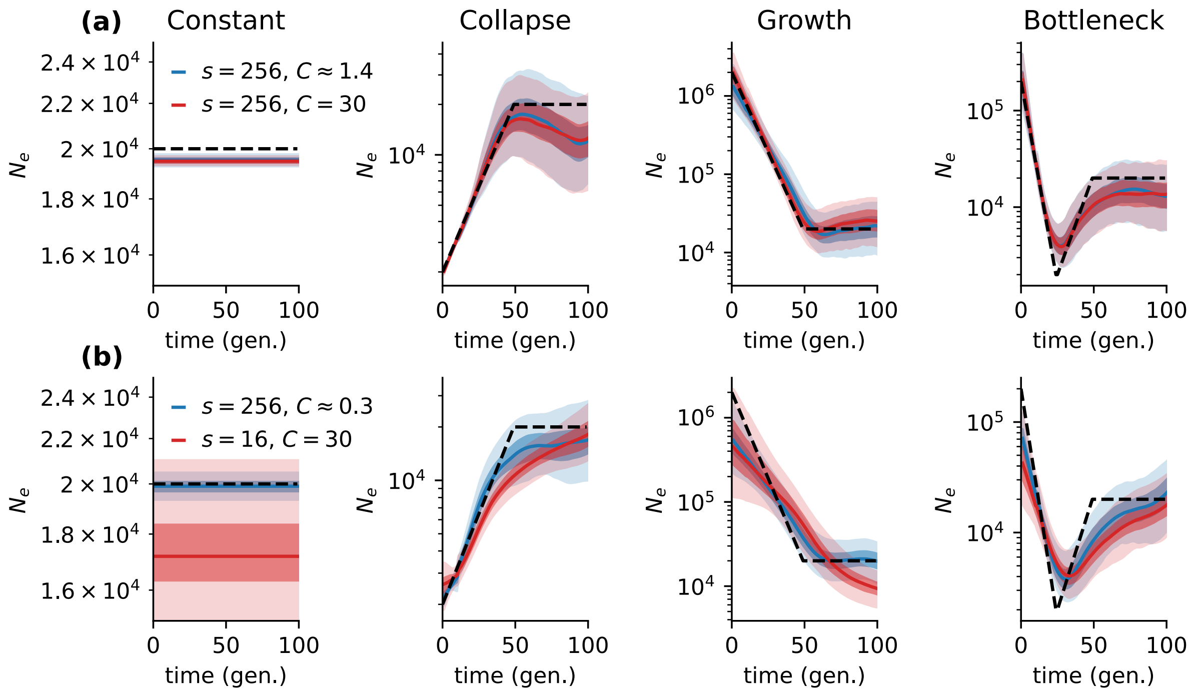

Supplementary Figure 12. **Effect of coverage and sample size.** (a) Output of HapNe-LD on simulated aDNA for 256 individuals, with $m = 0$ ($C \approx 30$) and $m = 0.25$ ($C \approx 1.4$). (b) Output of HapNe-LD on simulated aDNA for 16 individuals with $m = 0$ ($C \approx 30$) and 256 individuals with $m = 0.75$ ($C \approx 0.3$).

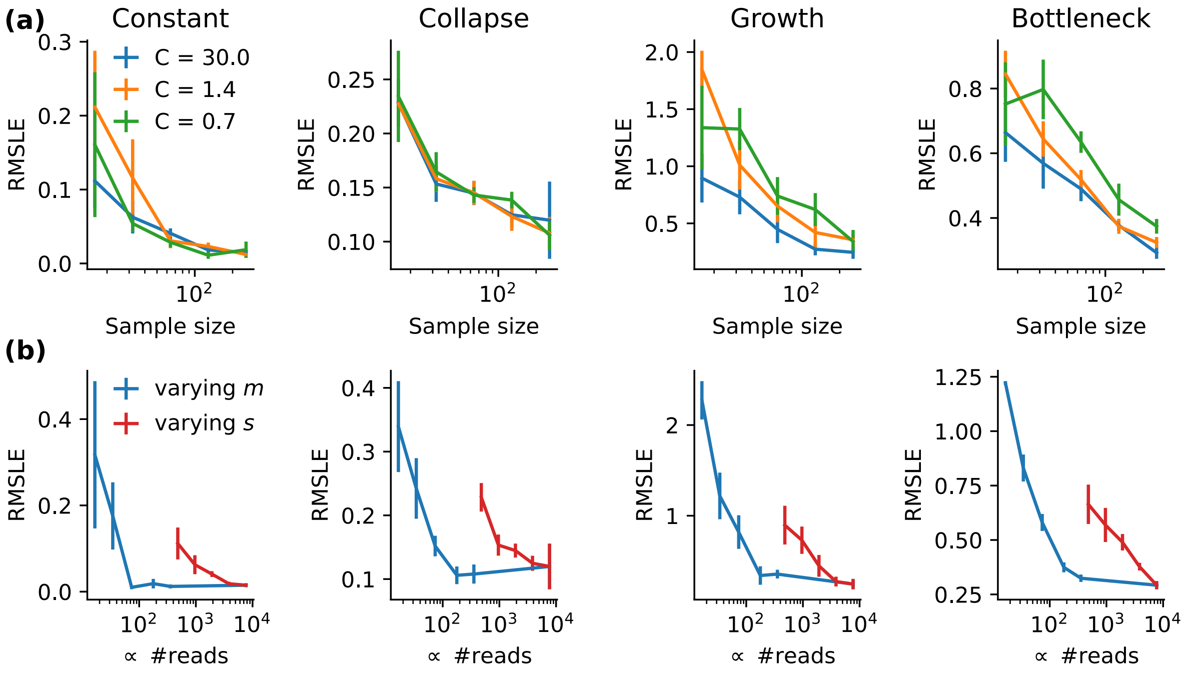

Supplementary Figure 13. **Accuracy of HapNe-LD as a function of sample size and coverage.** (a) RMSLE for HapNe-LD as a function of sample size for three different levels of coverage (line color) and different demographic models (column). The different levels of coverage, 30×, 1.4× and 0.7×, approximately correspond to $m = 0$, $m = 0.25$ and $m = 0.5$, respectively (see Methods). (b) Comparison of the RMSLE while keeping the number of samples constant ($s = 256$) and decreasing coverage (blue line), compared to the RMSLE obtained while keeping the coverage constant at 30×, while decreasing the sample size.

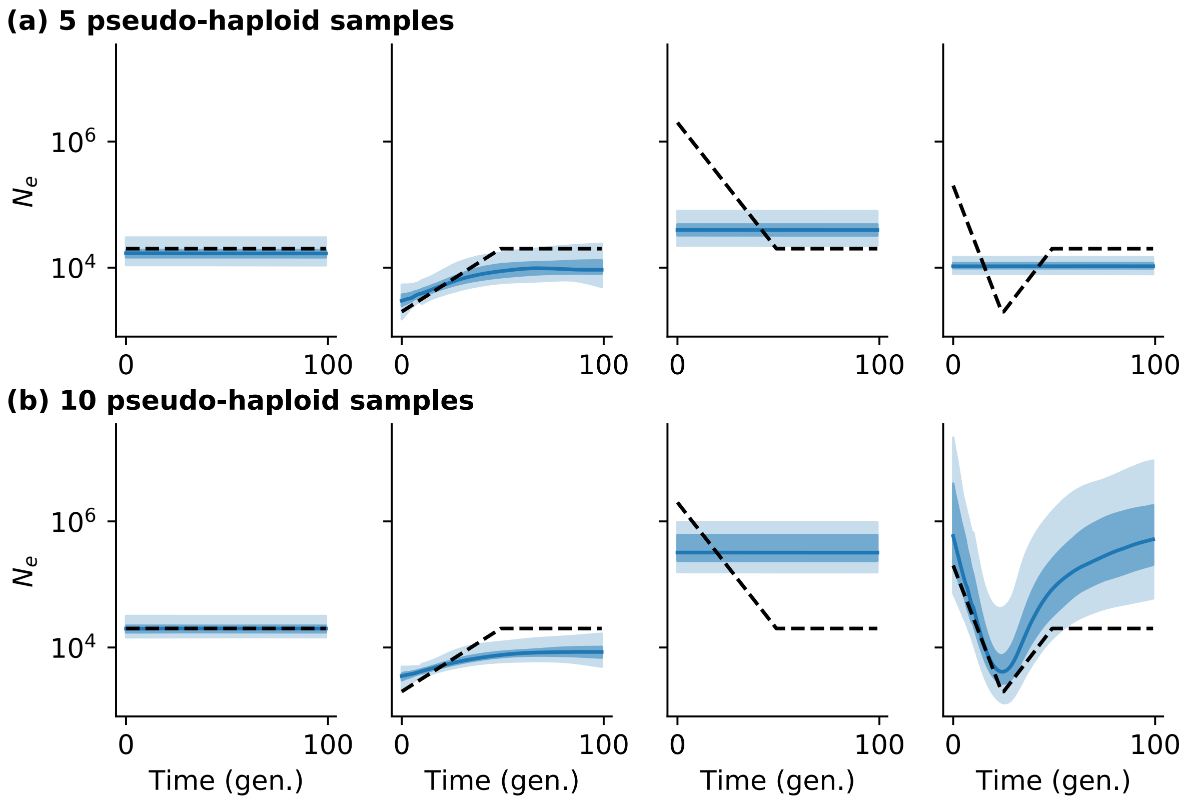

Supplementary Figure 14. **HapNe-LD inference with low sample sizes.** (a) Output of HapNe-LD on simulated aDNA for 5 (a) and 10 (b) individuals.

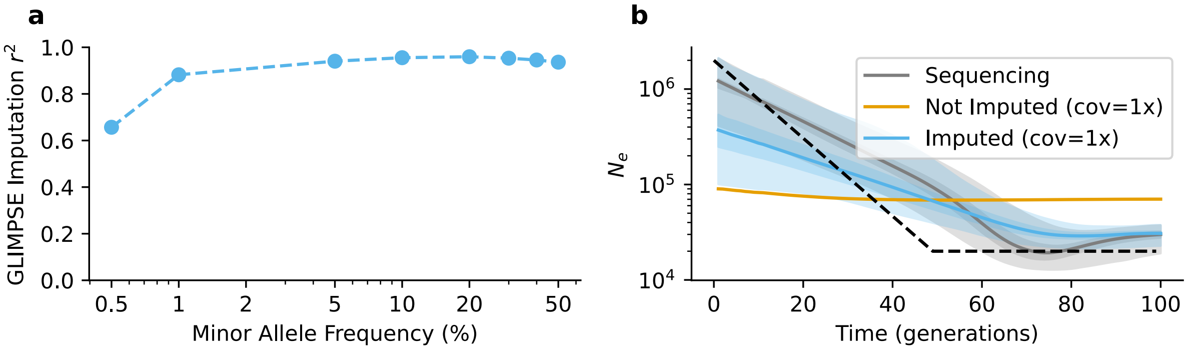

Supplementary Figure 15. **HapNe-LD analysis of imputed synthetic ancient samples.** We simulated 32 ancient individuals genotyped with a coverage of 1x, which we imputed using a reference panel of 200 diploid individuals (see Methods). (a) Imputation quality, measured as the r^2 of true and imputed genotypes at heterozygous sites for different minor allele frequencies. Imputed loci with MAF larger than 25% had a genotyping error rate of 2.9%.(b) Inference based on ground truth genotypes of the 32 individuals (Sequencing), aDNA-like pseudo-diploid data (Not imputed), and imputed data (Imputed). Confidence intervals for the aDNA-like analysis are omitted to improve readability.

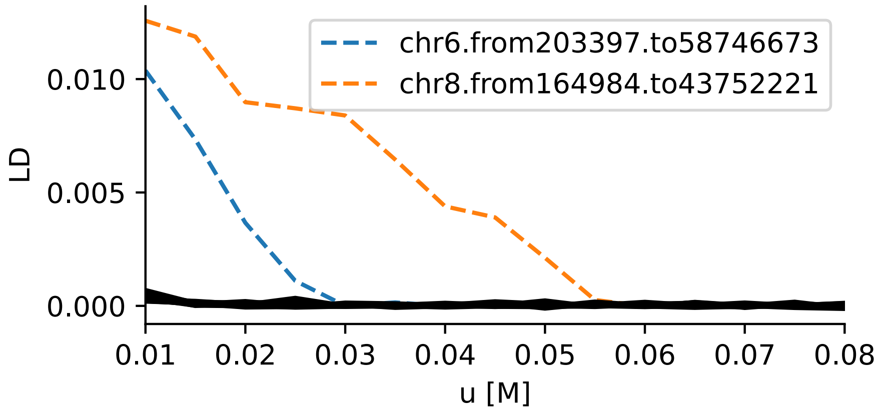

Supplementary Figure 16. **Filtering of high LD regions.** The LD at different distances u (in Morgans, M) was computed by randomly selecting individuals from the UK Biobank. Unusually elevated LD was observed in the HLA region on Chromosome 6 (blue line) and on Chromosome 8 (orange line), corresponding to a known large inversion polymorphism.

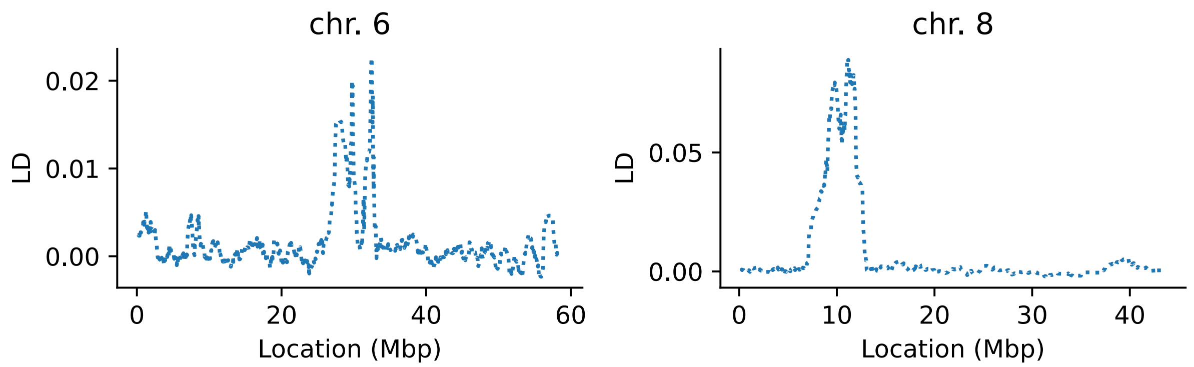

Supplementary Figure 17. **LD of SNPs in regions excluded from the GBR population in the 1kgp analysis.** The average LD of each SNP with loci located at a genetic distance of 2cM is shown for two regions excluded from the analysis by the HapNe filter. Unusually elevated LD was observed in the HLA region on Chromosome 6 (left) and on a known large inversion polymorphism in Chromosome 8 (right).

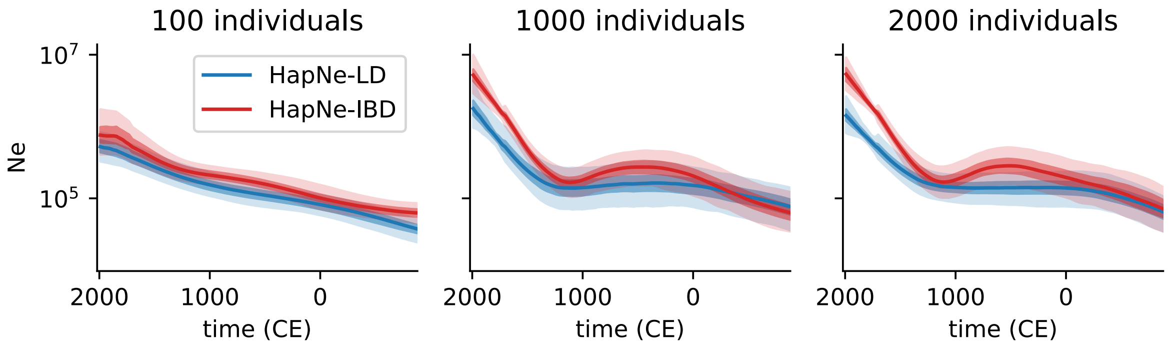

Supplementary Figure 18. **Downsampling analysis for the Glasgow postcode in the UK Biobank.** Effective population size inferred using unrelated individuals with self-reported white British ancestry whose birth location is in the Glasgow (G) postcode area. The numbers above each plot correspond to the sample size used in each analysis.

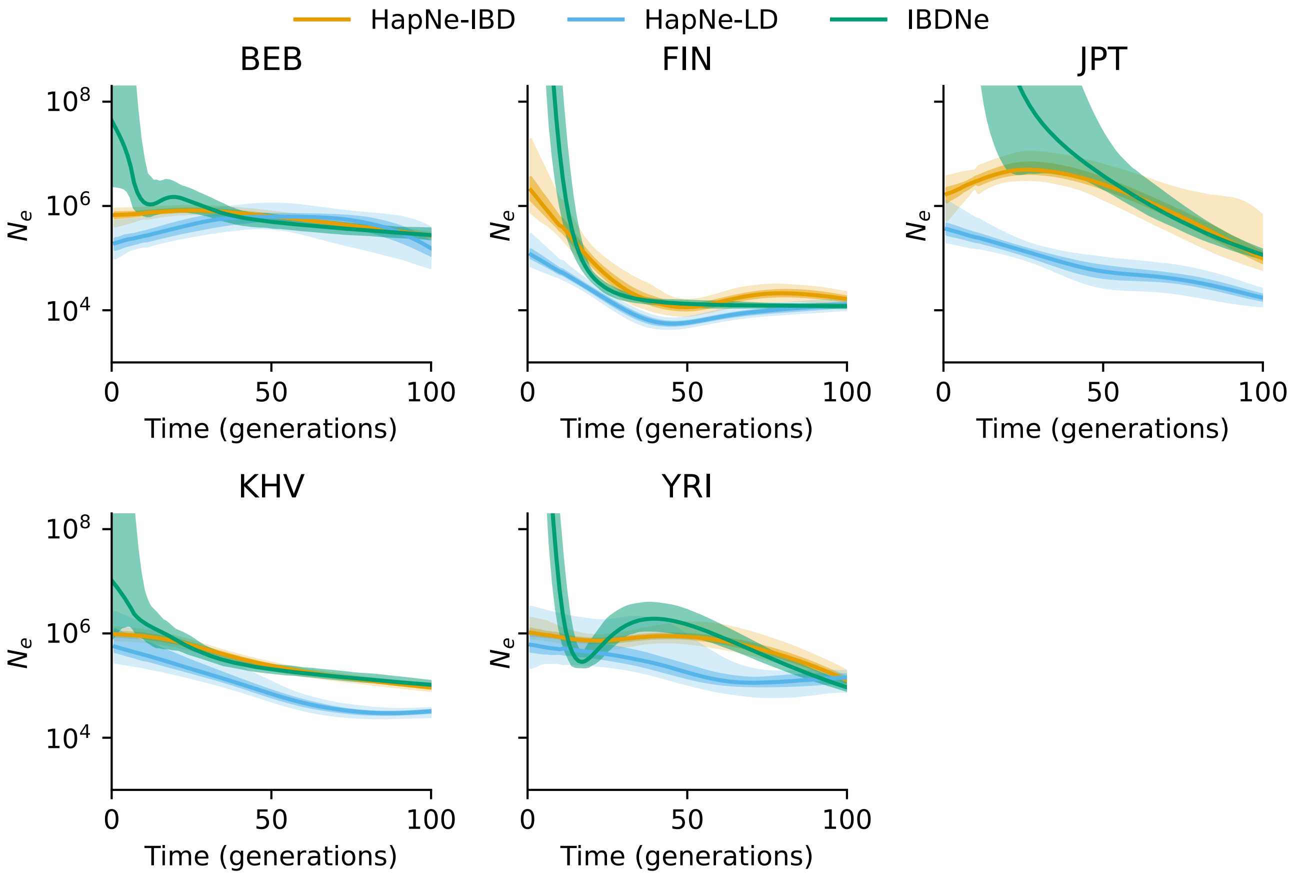

Supplementary Figure 19. **Results for the five populations of the** 1,000 **Genomes Project meeting filtering criteria and not flagged by HapNe-LD.** Populations (sample sizes) include BEB (s = 86), FIN (s = 99), JPT (s = 104), KHV (s = 99), and YRI (s = 108). IBD segments used as input of HapNe-IBD and IBDNe were detected using HapIBD and post-processed to merge adjacent segments (see Methods). The y-axis was truncated from 10^{63}, the upper confidence interval of IBDNe for the JPT population at $t = 0$, to 2×10^8 for readability.

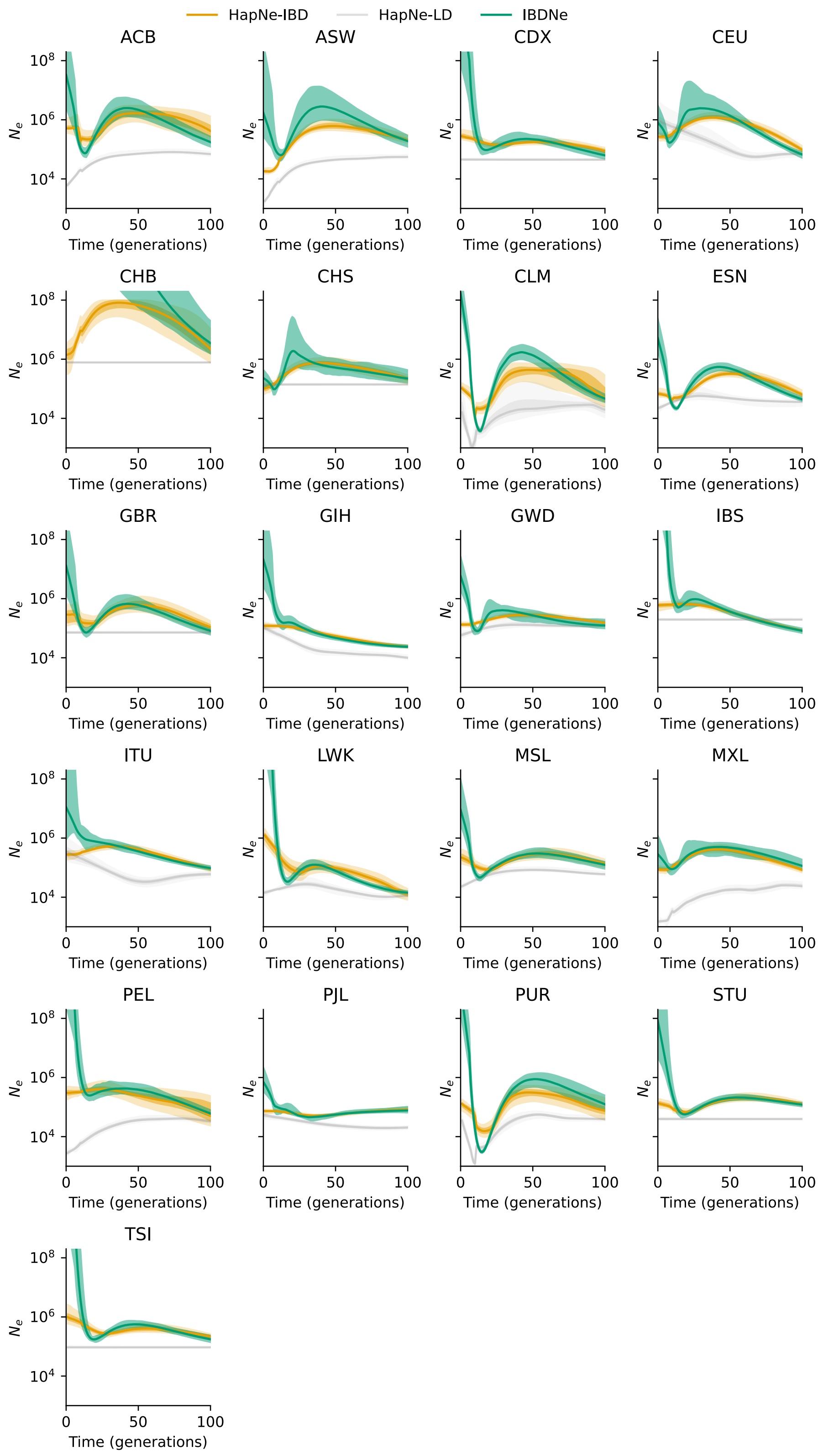

Supplementary Figure 20. **Results for the populations of the** 1,000 **Genomes Project flagged by HapNe-LD**. In this setting, the output of HapNe-LD may be biased. Populations (sample sizes) include ACB ($s = 96$), ASW ($s = 61$), CDX ($s = 93$), CEU ($s = 99$), CHB ($s = 103$), CHS ($s = 105$), CLM ($s = 94$), ESN ($s = 99$), GBR ($s = 91$), GIH ($s = 103$), GWD ($s = 113$), IBD ($s = 107$), ITU ($s = 102$), LWK ($s = 99$), MSL ($s = 85$), MXL ($s = 64$), PEL ($s = 85$), PJL ($s = 96$), PUR ($s = 104$), STU ($s = 102$), and TSI ($s = 107$). IBD segments used as input to HapNe-IBD and IBDNe were detected using HapIBD and post-processed to merge adjacent segments (see Methods). The y-axis was truncated from 10^{55}, the upper confidence interval of IBDNe for the CHB population at $t = 0$, to 2×10^8 for readability.

1.5 Supplementary Tables

**Population**	**s**	**Avg. Cov.**	**Date From (bp)**	**Date to (bp)**	$-\log_{10}$ **pval**
Arras in Pocklington	24	2.94	2175	2202	0.54
Hampshire MIA(-LIA)	14	1.98	2114	2225	0.08
South England MIA(-LIA)	49	2.88	2022	2227	1.00
Viking Norway	22	1.50	950	1100	1.51
Viking Gotland	28	1.45	975	975	3.52
Caribbean Ceramic	71	2.74	510	801	inf
Dominican SE coast Ceramic	18	3.08	849	1150	inf

Supplementary Table 1. **Further information on populations analyzed in Figure 4 of the main text.**
Sample size s, average coverage, estimated age of the most recent and distant samples (given in years before 1950), and approximate p-value for the CCLD test for each analyzed ancient population. The approximate p-value was computed using a two-sided t-test for the observed CCLD being centered at the value expected from the sample size and coverage. Note that, due to dependencies across pairs of regions, this is an approximate p-value, making this a conservative filtering criterion.

Master ID	Publication	Group ID	Source
I5505	PattersonNature2022[27]	England_EastYorkshire_MIA_LIA	Publication
I12414	PattersonNature2022	England_EastYorkshire_MIA_LIA	Publication
I12413	PattersonNature2022	England_EastYorkshire_MIA_LIA	Publication
I12415	PattersonNature2022	England_EastYorkshire_MIA_LIA	Publication
I12411	PattersonNature2022	England_EastYorkshire_MIA_LIA	Publication
I11034	PattersonNature2022	England_EastYorkshire_MIA_LIA	Publication
I13759	PattersonNature2022	England_EastYorkshire_MIA_LIA	Publication
I14104	PattersonNature2022	England_EastYorkshire_MIA_LIA	Publication
I14101	PattersonNature2022	England_EastYorkshire_MIA_LIA	Publication
I14099	PattersonNature2022	England_EastYorkshire_MIA_LIA	Publication
I13753	PattersonNature2022	England_EastYorkshire_MIA_LIA	Publication
I13756	PattersonNature2022	England_EastYorkshire_MIA_LIA	Publication
I13757	PattersonNature2022	England_EastYorkshire_MIA_LIA	Publication
I13754	PattersonNature2022	England_EastYorkshire_MIA_LIA	Publication
I13760	PattersonNature2022	England_EastYorkshire_MIA_LIA	Publication
I14107	PattersonNature2022	England_EastYorkshire_MIA_LIA	Publication
I13755	PattersonNature2022	England_EastYorkshire_MIA_LIA	Publication
I5510	PattersonNature2022	England_EastYorkshire_MIA_LIA	Publication
I14103	PattersonNature2022	England_EastYorkshire_MIA_LIA	Publication
I5506	PattersonNature2022	England_EastYorkshire_MIA_LIA	Publication
I14105	PattersonNature2022	England_EastYorkshire_MIA_LIA	Publication
I5508	PattersonNature2022	England_EastYorkshire_MIA_LIA	Publication
I14102	PattersonNature2022	England_EastYorkshire_MIA_LIA	Publication
I5511	PattersonNature2022	England_EastYorkshire_MIA_LIA	Publication

Supplementary Table 2. **Samples used in the Arras analysis** Genotypes were downloaded from published supplementary materials.

Master ID	Publication	Group ID	Source
I17262	PattersonNature2022	England_MIA_LIA	Publication
I20987	PattersonNature2022	England_MIA_LIA	Publication
I20985	PattersonNature2022	England_MIA_LIA	Publication
I20983	PattersonNature2022	England_MIA_LIA	Publication
I20986	PattersonNature2022	England_MIA_LIA	Publication
I20982	PattersonNature2022	England_MIA_LIA	Publication
I20984	PattersonNature2022	England_MIA_LIA	Publication
I17261	PattersonNature2022	England_MIA	Publication
I17263	PattersonNature2022	England_MIA_LIA	Publication
I20989	PattersonNature2022	England_MIA_LIA	Publication
I20990	PattersonNature2022	England_MIA	Publication
I17267	PattersonNature2022	England_MIA_LIA	Publication
I20988	PattersonNature2022	England_MIA_LIA	Publication
I17264	PattersonNature2022	England_MIA_LIA	Publication

Supplementary Table 3. **Samples used in the Hampshire analysis** Genotypes were downloaded from published supplementary materials.

Master ID	Publication	Group ID	Source
I11145	PattersonNature2022[27]	England_LIA	Publication
I19869	PattersonNature2022	England_LIA_daughter.I19870	Publication
I16458	PattersonNature2022	England_MIA_LIA	Publication
I16457	PattersonNature2022	England_MIA_LIA	Publication
I16450	PattersonNature2022	England_MIA_LIA	Publication
I17017	PattersonNature2022	England_LIA_highEEF	Publication
I21308	PattersonNature2022	England_MIA_LIA	Publication
I11142	PattersonNature2022	England_LIA	Publication
I27379	PattersonNature2022	England_LIA	Publication
I21311	PattersonNature2022	England_MIA_LIA	Publication
I16601	PattersonNature2022	England_MIA_LIA	Publication
I11992	PattersonNature2022	England_MIA_LIA	Publication
I21312	PattersonNature2022	England_MIA_LIA	Publication
I17263	PattersonNature2022	England_MIA_LIA	Publication
I21310	PattersonNature2022	England_MIA_LIA	Publication
I11991	PattersonNature2022	England_MIA_LIA	Publication
I21307	PattersonNature2022	England_MIA_LIA	Publication
I13726	PattersonNature2022	England_MIA_LIA	Publication
I11143	PattersonNature2022	England_MIA_LIA	Publication
I21309	PattersonNature2022	England_MIA_LIA	Publication
I21313	PattersonNature2022	England_MIA_LIA	Publication
I20989	PattersonNature2022	England_MIA_LIA	Publication
I17262	PattersonNature2022	England_MIA_LIA	Publication
I20987	PattersonNature2022	England_MIA_LIA	Publication
I20985	PattersonNature2022	England_MIA_LIA	Publication

I20983	PattersonNature2022	England_MIA_LIA	Publication
I20986	PattersonNature2022	England_MIA_LIA	Publication
I20982	PattersonNature2022	England_MIA_LIA	Publication
I20984	PattersonNature2022	England_MIA_LIA	Publication
I19657	PattersonNature2022	England_MIA_LIA	Publication
I19855	PattersonNature2022	England_MIA_LIA	Publication
I19854	PattersonNature2022	England_MIA_LIA	Publication
I11993	PattersonNature2022	England_MIA_LIA	Publication
I11994	PattersonNature2022	England_MIA_LIA	Publication
I12792	PattersonNature2022	England_MIA_LIA_mother.I12793	Publication
I20990	PattersonNature2022	England_MIA	Publication
I19912	PattersonNature2022	England_MIA	Publication
I13680	PattersonNature2022	England_MIA	Publication
I17261	PattersonNature2022	England_MIA	Publication
I14863	PattersonNature2022	England_MIA	Publication
I17267	PattersonNature2022	England_MIA_LIA	Publication
I20988	PattersonNature2022	England_MIA_LIA	Publication
I17264	PattersonNature2022	England_MIA_LIA	Publication
I14866	PattersonNature2022	England_MIA	Publication
I17016	PattersonNature2022	England_MIA	Publication
I14859	PattersonNature2022	England_MIA	Publication
I17015	PattersonNature2022	England_MIA	Publication
I19909	PattersonNature2022	England_MIA	Publication
I17014	PattersonNature2022	England_MIA	Publication

Supplementary Table 4. **Samples used in the South England MIA-LIA analysis** Genotypes were downloaded from published supplementary materials.

Master ID	Publication	Group ID	Source
VK387	MargaryanWillerslevNature2020[28]	Norway_Viking.SG	V50[29]
VK414	MargaryanWillerslevNature2020	Norway_Viking.SG	V50
VK530	MargaryanWillerslevNature2020	Norway_Viking_o2.SG	V50
VK386	MargaryanWillerslevNature2020	Norway_Viking.SG	V50
VK389	MargaryanWillerslevNature2020	Norway_Viking.SG	V50
VK393	MargaryanWillerslevNature2020	Norway_Viking.SG	V50
VK394	MargaryanWillerslevNature2020	Norway_Viking.SG	V50
VK422	MargaryanWillerslevNature2020	Norway_Viking.SG	V50
VK515	MargaryanWillerslevNature2020	Norway_Viking.SG	V50
VK516	MargaryanWillerslevNature2020	Norway_Viking.SG	V50
VK520	MargaryanWillerslevNature2020	Norway_Viking.SG	V50
VK524	MargaryanWillerslevNature2020	Norway_Viking.SG	V50
VK415	MargaryanWillerslevNature2020	Norway_Viking.SG	V50
VK420	MargaryanWillerslevNature2020	Norway_Viking.SG	V50
VK448	MargaryanWillerslevNature2020	Norway_Viking.SG	V50
VK547	MargaryanWillerslevNature2020	Norway_Viking.SG	V50
VK518	MargaryanWillerslevNature2020	Norway_Viking_o1.SG	V50

VK392	MargaryanWillerslevNature2020	Norway_Viking.SG	V50
VK417	MargaryanWillerslevNature2020	Norway_Viking.SG	V50
VK525	MargaryanWillerslevNature2020	Norway_Viking.SG	V50
VK526	MargaryanWillerslevNature2020	Norway_Viking.SG	V50
VK548	MargaryanWillerslevNature2020	Norway_Viking.SG	V50

Supplementary Table 5. **Samples used in the Norway Viking analysis**. Genotypes were downloaded from V50 of the Allen ancient data resource.[29]

Master ID	Publication	Group ID	Source
VK58	MargaryanWillerslevNature2020[28]	Sweden_Viking.SG	V50[29]
VK429	MargaryanWillerslevNature2020	Sweden_Viking.SG	V50
VK433	MargaryanWillerslevNature2020	Sweden_Viking.SG	V50
VK455	MargaryanWillerslevNature2020	Sweden_Viking.SG	V50
VK456	MargaryanWillerslevNature2020	Sweden_Viking.SG	V50
VK56	MargaryanWillerslevNature2020	Sweden_Viking.SG	V50
VK64	MargaryanWillerslevNature2020	Sweden_Viking.SG	V50
VK60	MargaryanWillerslevNature2020	Sweden_Viking.SG	V50
VK432	MargaryanWillerslevNature2020	Sweden_Viking.SG	V50
VK460	MargaryanWillerslevNature2020	Sweden_Viking.SG	V50
VK461	MargaryanWillerslevNature2020	Sweden_Viking.SG	V50
VK463	MargaryanWillerslevNature2020	Sweden_Viking.SG	V50
VK434	MargaryanWillerslevNature2020	Sweden_Viking.SG	V50
VK431	MargaryanWillerslevNature2020	Sweden_Viking.SG	V50
VK475	MargaryanWillerslevNature2020	Sweden_Viking.SG	V50
VK468	MargaryanWillerslevNature2020	Sweden_Viking.SG	V50
VK50	MargaryanWillerslevNature2020	Sweden_Viking.SG	V50
VK479	MargaryanWillerslevNature2020	Sweden_Viking.SG	V50
VK474	MargaryanWillerslevNature2020	Sweden_Viking.SG	V50
VK478	MargaryanWillerslevNature2020	Sweden_Viking.SG	V50
VK473	MargaryanWillerslevNature2020	Sweden_Viking.SG	V50
VK477	MargaryanWillerslevNature2020	Sweden_Viking.SG	V50
VK53	MargaryanWillerslevNature2020	Sweden_Viking.SG	V50
VK51	MargaryanWillerslevNature2020	Sweden_Viking.SG	V50
VK232	MargaryanWillerslevNature2020	Sweden_Viking.SG	V50
VK48	MargaryanWillerslevNature2020	Sweden_Viking.SG	V50
VK454	MargaryanWillerslevNature2020	Sweden_Viking.SG	V50
VK452	MargaryanWillerslevNature2020	Sweden_Viking.SG	V50

Supplementary Table 6. **Samples used in the Gotland Viking analysis**. Genotypes were downloaded from V50 of the Allen ancient data resource.[29]

Master ID	Publication	Group ID	Source
I15109	FernandesSirakNature2020[30]	Dominican_Atajadizo_Ceramic	V50[29]
I15108	FernandesSirakNature2020	Dominican_Atajadizo_Ceramic	V50
CDE003	NagelePosthScience2020[31]	Cuba_CuevaEsqueletos_Ceramic	V50

I15667	FernandesSirakNature2020	Dominican_LaCaleta_Ceramic.SG	V50
I13206	FernandesSirakNature2020	Dominican_JuanDolio_Ceramic	V50
I15667	FernandesSirakNature2020	Dominican_LaCaleta_Ceramic	V50
I17901	FernandesSirakNature2020	Dominican_Atajadizo_Ceramic	V50
I15962	FernandesSirakNature2020	Dominican_LaCaleta_Ceramic.SG	V50
I15962	FernandesSirakNature2020	Dominican_LaCaleta_Ceramic	V50
I17908	FernandesSirakNature2020	Dominican_Atajadizo_Ceramic	V50
I13207	FernandesSirakNature2020	Dominican_JuanDolio_Ceramic	V50
I17900	FernandesSirakNature2020	Dominican_Atajadizo_Ceramic	V50
ELM001	NagelePosthScience2020	Cuba_ElMorrillo_Ceramic	V50
I13199	FernandesSirakNature2020	Dominican_JuanDolio_Ceramic	V50
I15972	FernandesSirakNature2020	Dominican_LaCaleta_Ceramic	V50
I14992	FernandesSirakNature2020	Dominican_LosMuertos_Ceramic	V50
I17907	FernandesSirakNature2020	Dominican_Atajadizo_Ceramic	V50
I14883	FernandesSirakNature2020	Bahamas_SouthAndros_Ceramic.SG	V50
I14880	FernandesSirakNature2020	Bahamas_SouthAndros_Ceramic.SG	V50
I14880	FernandesSirakNature2020	Bahamas_SouthAndros_Ceramic	V50
I14881	FernandesSirakNature2020	Bahamas_SouthAndros_Ceramic	V50
I15668	FernandesSirakNature2020	Dominican_LaCaleta_Ceramic	V50
I13201	FernandesSirakNature2020	Dominican_JuanDolio_Ceramic	V50
I7970	FernandesSirakNature2020	Dominican_LaUnion_Ceramic	V50
I13195	FernandesSirakNature2020	Dominican_ElSoco_Ceramic	V50
I14923	FernandesSirakNature2020	Bahamas_AbacoIsl_Ceramic	V50
I15107	FernandesSirakNature2020	Dominican_Atajadizo_Ceramic	V50
I7969	FernandesSirakNature2020	Dominican_LaUnion_Ceramic	V50
I15111	FernandesSirakNature2020	Dominican_Atajadizo_Ceramic	V50
I13738	FernandesSirakNature2020	Bahamas_LongIsl_Ceramic_published	V50
I13739	FernandesSirakNature2020	Bahamas_LongIsl_Ceramic_published	V50
I14991	FernandesSirakNature2020	Dominican_LomaPerenal_Ceramic	V50
I15591	FernandesSirakNature2020	Dominican_LaCaleta_Ceramic	V50
I7971	FernandesSirakNature2020	Dominican_LaUnion_Ceramic	V50
I14882	FernandesSirakNature2020	Bahamas_SouthAndros_Ceramic.SG	V50
I14882	FernandesSirakNature2020	Bahamas_SouthAndros_Ceramic	V50
I15973	FernandesSirakNature2020	Dominican_LaCaleta_Ceramic	V50
I8118	FernandesSirakNature2020	Dominican_ElSoco_Ceramic	V50
I14879	FernandesSirakNature2020	Bahamas_SouthAndros_Ceramic.SG	V50
I14879	FernandesSirakNature2020	Bahamas_SouthAndros_Ceramic	V50
I14879	FernandesSirakNature2020	Bahamas_SouthAndros_Ceramic.SG	V50
LAV010	NagelePosthScience2020	StLucia_Lavoutte_Ceramic	V50
I13208	FernandesSirakNature2020	Dominican_JuanDolio_Ceramic	V50
I17902	FernandesSirakNature2020	Dominican_Atajadizo_Ceramic	V50
I13560	FernandesSirakNature2020	Bahamas_SouthAndros_Ceramic_published	V50
PDI008	NagelePosthScience2020	PuertoRico_PasodelIndio_Ceramic	V50
LAV003	NagelePosthScience2020	StLucia_Lavoutte_Ceramic	V50
I15082	FernandesSirakNature2020	Dominican_LaCaleta_Ceramic	V50

I16175	FernandesSirakNature2020	Dominican_LaCaleta_Ceramic	V50
I13196	FernandesSirakNature2020	Dominican_JuanDolio_Ceramic_father.or.son.I23524	V50
LAV002	NagelePosthScience2020	StLucia_Lavoutte_Ceramic	V50
I8549	FernandesSirakNature2020	Dominican_Andres_Ceramic	V50
I13192	FernandesSirakNature2020	Dominican_ElSoco_Ceramic	V50
I16176	FernandesSirakNature2020	Dominican_LaCaleta_Ceramic	V50
I14990	FernandesSirakNature2020	Dominican_EdilioCruz_Ceramic	V50
I13323	FernandesSirakNature2020	PuertoRico_SantaElena_Ceramic	V50
I15112	FernandesSirakNature2020	Dominican_Atajadizo_Ceramic	V50
I15106	FernandesSirakNature2020	Dominican_Atajadizo_Ceramic	V50
I14994	FernandesSirakNature2020	Dominican_LosCorniel_Ceramic	V50
I15105	FernandesSirakNature2020	Dominican_Atajadizo_Ceramic	V50
I13190	FernandesSirakNature2020	Dominican_ElSoco_Ceramic	V50
LAV006	NagelePosthScience2020	StLucia_Lavoutte_Ceramic	V50
LAV004	NagelePosthScience2020	StLucia_Lavoutte_Ceramic	V50
I13318	FernandesSirakNature2020	Bahamas_CrookedIsl_Ceramic	V50
I13321	FernandesSirakNature2020	Bahamas_EleutheraIsl_Ceramic	V50
I13319	FernandesSirakNature2020	Bahamas_CrookedIsl_Ceramic	V50
I13737	FernandesSirakNature2020	Bahamas_LongIsl_Ceramic	V50
I13189	FernandesSirakNature2020	Dominican_ElSoco_Ceramic	V50
I15966	FernandesSirakNature2020	Dominican_LaCaleta_Ceramic	V50
I18300	FernandesSirakNature2020	Dominican_Atajadizo_Ceramic	V50
PDI011	NagelePosthScience2020	PuertoRico_PasodelIndio_Ceramic	V50

Supplementary Table 7. **Samples used in the Caribbean Ceramic analysis**. Genotypes were downloaded from V50 of the Allen ancient data resource.[29]

Master ID	Publication	Group ID	Source
I8547	FernandesSirakNature2020	Dominican_Andres_Ceramic	V50
I15975	FernandesSirakNature2020	Dominican_LaCaleta_Ceramic	V50
I15081	FernandesSirakNature2020	Dominican_LaCaleta_Ceramic	V50
I15592	FernandesSirakNature2020	Dominican_LaCaleta_Ceramic	V50
I15672	FernandesSirakNature2020	Dominican_LaCaleta_Ceramic	V50
I15968	FernandesSirakNature2020	Dominican_LaCaleta_Ceramic.SG	V50
I16519	FernandesSirakNature2020	Dominican_LaCaleta_Ceramic	V50
I15978	FernandesSirakNature2020	Dominican_LaCaleta_Ceramic	V50
I15969	FernandesSirakNature2020	Dominican_LaCaleta_Ceramic	V50
I20527	FernandesSirakNature2020	Dominican_ElSoco_Ceramic.SG	V50
I20527	FernandesSirakNature2020	Dominican_ElSoco_Ceramic	V50
I15976	FernandesSirakNature2020	Dominican_LaCaleta_Ceramic	V50
I15682	FernandesSirakNature2020	Dominican_LaCaleta_Ceramic	V50
I12347	FernandesSirakNature2020	Dominican_ElSoco_Ceramic	V50
I12344	FernandesSirakNature2020	Dominican_ElSoco_Ceramic	V50
I12350	FernandesSirakNature2020	Dominican_ElSoco_Ceramic	V50
I12341	FernandesSirakNature2020	Dominican_ElSoco_Ceramic	V50

I8121	FernandesSirakNature2020	Dominican_ElSoco_Ceramic_published	V50

Supplementary Table 8. **Samples used in the South East Coast Dominican Republic Ceramic analysis**. Genotypes were downloaded from V50 of the Allen ancient data resource.[29]

Supplementary References

[1] Marjoram, P. & Wall, J. D. Fast "coalescent" simulation. *BMC Genetics* **7** (2006).

[2] Palamara, P. F., Lencz, T., Darvasi, A. & Pe'er, I. Length distributions of identity by descent reveal fine-scale demographic history. *American Journal of Human Genetics* **91**, 809–822 (2012).

[3] Harris, K. & Nielsen, R. Inferring demographic history from a spectrum of shared haplotype lengths. *PLoS Genetics* **9** (2013).

[4] Ralph, P. & Coop, G. The geography of recent genetic ancestry across europe. *PLoS Biology* **11**, 1001555 (2013).

[5] Schiffels, S. & Durbin, R. Inferring human population size and separation history from multiple genome sequences. *Nature Genetics* **46**, 919–925 (2014).

[6] Palamara, P. F. *Population genetics of identity by descent* (Columbia University, 2014).

[7] Carmi, S., Wilton, P. R., Wakeley, J. & Pe'er, I. A renewal theory approach to IBD sharing. *Theoretical Population Biology* **97**, 35–48 (2014).

[8] Browning, S. R. & Browning, B. L. Accurate non-parametric estimation of recent effective population size from segments of identity by descent. *American Journal of Human Genetics* **97**, 404–418 (2015).

[9] Palamara, P. F. *et al.* Leveraging distant relatedness to quantify human mutation and gene-conversion rates. *The American Journal of Human Genetics* **97**, 775–789 (2015).

[10] Wilton, P. R., Carmi, S. & Hobolth, A. The smc' is a highly accurate approximation to the ancestral recombination graph. *Genetics* **200**, 343–355 (2015).

[11] Biddanda, A., Steinrücken, M. & Novembre, J. Properties of 2-locus genealogies and linkage disequilibrium in temporally structured samples. *Genetics* **221** (2022).

[12] Kingman, J. The coalescent. *Stochastic Processes and their Applications* **13**, 235–248 (1982).

[13] Wiuf, C. & Hein, J. Recombination as a point process along sequences. *Theoretical population biology* **55**, 248–259 (1999).

[14] Eriksson, A., Mahjani, B. & Mehlig, B. Sequential markov coalescent algorithms for population models with demographic structure. *Theoretical Population Biology* **76**, 84–91 (2009).

[15] McVean, G. A. & Cardin, N. J. Approximating the coalescent with recombination. *Philosophical Transactions of the Royal Society B: Biological Sciences* **360**, 1387–1393 (2005).

[16] Sved, J. Linkage disequilibrium and homozygosity of chromosome segments in finite populations. *Theoretical Population Biology* **2** (1971).

[17] Davison, A. C. *Statistical Models* (Cambridge University Press, Cambridge, 2003).

[18] Hudson, R. R. THE SAMPLING DISTRIBUTION OF LINKAGE DISEQUILIBRIUM UNDER AN INFINITE ALLELE MODEL WITHOUT SELECTION. *Genetics* **109**, 611–631 (1985).

[19] Wang, K., Mathieson, I., O'Connell, J. & Schiffels, S. Tracking human population structure through time from whole genome sequences. *PLOS Genetics* **16**, e1008552 (2020).

[20] Slatkin, M. Inbreeding coefficients and coalescence times. *Genetical Research* **58**, 167–175 (1991).

[21] Myers, S., Fefferman, C. & Patterson, N. Can one learn history from the allelic spectrum? *Theoretical Population Biology* **73**, 342–348 (2008).

[22] Bhaskar, A. & Song, Y. S. Descartes' rule of signs and the identifiability of population demographic models from genomic variation data. *The Annals of Statistics* **42** (2014).

[23] Terhorst, J., Kamm, J. A. & Song, Y. S. Robust and scalable inference of population history from hundreds of unphased whole genomes. *Nature Genetics* **49** (2017).

[24] Virtanen, P. *et al.* SciPy 1.0: Fundamental Algorithms for Scientific Computing in Python. *Nature Methods* **17**, 261–272 (2020).

[25] Hinch, A. G. *et al.* The landscape of recombination in african americans. *Nature* **476**, 170–175 (2011).

[26] Nelis, M. *et al.* Genetic structure of europeans: A view from the north–east. *PLoS ONE* **4**, e5472 (2009).

[27] Patterson, N. *et al.* Large-scale migration into britain during the middle to late bronze age. *Nature* (2021).

[28] Margaryan, A. *et al.* Population genomics of the viking world. *Nature* **585**, 390–396 (2020).

[29] Mallick, S. & Reich, D. The allen ancient dna resource (aadr): A curated compendium of ancient human genomes, harvard dataverse, v50.0 data release [october 10 2021] (2021).

[30] Fernandes, D. M. *et al.* A genetic history of the pre-contact caribbean. *Nature* **590**, 103–110 (2021).

[31] Nägele, K. *et al.* Genomic insights into the early peopling of the caribbean. *Science* **369**, 456–460 (2020).
