# Supplementary material for: Haplotype-based inference of recent effective population size in modern and ancient DNA samples
Source: Nat Commun. 2023 Dec 1;14:7945. doi: 10.1038/s41467-023-43522-6 (PMC10692198; doi:10.1038/s41467-023-43522-6)
Supplement: Supplementary file 2 — Reporting Summary [file 41467_2023_43522_MOESM2_ESM.pdf]

Corresponding author(s): Pier Palamara

Last updated by author(s): Sep 22, 2023

Reporting Summary

Nature Portfolio wishes to improve the reproducibility of the work that we publish. This form provides structure for consistency and transparency in reporting. For further information on Nature Portfolio policies, see our Editorial Policies and the Editorial Policy Checklist.

Statistics

For all statistical analyses, confirm that the following items are present in the figure legend, table legend, main text, or Methods section.

n/a	Confirmed	
☐	☒	The exact sample size (*n*) for each experimental group/condition, given as a discrete number and unit of measurement
☐	☒	A statement on whether measurements were taken from distinct samples or whether the same sample was measured repeatedly
☐	☒	The statistical test(s) used AND whether they are one- or two-sided<br>*Only common tests should be described solely by name; describe more complex techniques in the Methods section.*
☐	☒	A description of all covariates tested
☐	☒	A description of any assumptions or corrections, such as tests of normality and adjustment for multiple comparisons
☐	☒	A full description of the statistical parameters including central tendency (e.g. means) or other basic estimates (e.g. regression coefficient) AND variation (e.g. standard deviation) or associated estimates of uncertainty (e.g. confidence intervals)
☐	☒	For null hypothesis testing, the test statistic (e.g. *F*, *t*, *r*) with confidence intervals, effect sizes, degrees of freedom and *P* value noted<br>*Give P values as exact values whenever suitable.*
☐	☒	For Bayesian analysis, information on the choice of priors and Markov chain Monte Carlo settings
☒	☐	For hierarchical and complex designs, identification of the appropriate level for tests and full reporting of outcomes
☐	☒	Estimates of effect sizes (e.g. Cohen's *d*, Pearson's *r*), indicating how they were calculated

Our web collection on statistics for biologists contains articles on many of the points above.

Software and code

Policy information about availability of computer code

Data collection	We used the following software to simulate synthetic data:<br>- ARGON version 0.1.160415 (palamaralab.github.io/software/argon/)<br>- msprime version 1.1.1 (tskit.dev/msprime)
Data analysis	The HapNe software package is freely available at palamaralab.github.io/software/hapne and https://pypi.org/project/hapne/.<br><br>We also used the following software:<br>plink1.9, pkink2.0, FastSMC (version 1.2), HapIBD (version 1.0, 23Apr20.f1a), RefinedIBD (version 17jan20), IBDNe (version 23Apr20.ae9), GONe (retrieved on Jun 22, 2021), Python3.7, Numpy (1.23.4), Pandas (1.51), Scipy (1.9.3), Numba(0.56.3), Matplotlib (3.4.3), Seaborn(0.12.2), Geopandas (0.12.2).

For manuscripts utilizing custom algorithms or software that are central to the research but not yet described in published literature, software must be made available to editors and reviewers. We strongly encourage code deposition in a community repository (e.g. GitHub). See the Nature Portfolio guidelines for submitting code & software for further information.

Data

Policy information about availability of data

All manuscripts must include a data availability statement. This statement should provide the following information, where applicable:

- Accession codes, unique identifiers, or web links for publicly available datasets
- A description of any restrictions on data availability
- For clinical datasets or third party data, please ensure that the statement adheres to our policy

Genomic data sets and annotations analyzed in this study include:

UK Biobank http://www.ukbiobank.ac.uk/
genetic maps ftp://1000genomes.ebi.ac.uk/vol1/ftp/technical/working/20110106_recombination_hotspots/
1000 Genomes Project phase three https://www.internationalgenome.org/data
Allen Ancient DNA Resource https://reich.hms.harvard.edu/allen-ancient-dna-resource-aadr-downloadable-genotypes-present-day-and-ancient-dna-data

Human research participants

Policy information about studies involving human research participants and Sex and Gender in Research.

Reporting on sex and gender	n/a
Population characteristics	n/a
Recruitment	n/a
Ethics oversight	n/a

Note that full information on the approval of the study protocol must also be provided in the manuscript.

Field-specific reporting

Please select the one below that is the best fit for your research. If you are not sure, read the appropriate sections before making your selection.

☒ Life sciences ☐ Behavioural & social sciences ☐ Ecological, evolutionary & environmental sciences

For a reference copy of the document with all sections, see nature.com/documents/nr-reporting-summary-flat.pdf

Life sciences study design

All studies must disclose on these points even when the disclosure is negative.

Sample size	For real data analysis in the UK Biobank, we included all 305,784 unrelated individuals of white British ancestry (as reported in Bycroft et al. Nature 2018) who had not withdrawn from the UK Biobank at the time of our analysis.<br><br>For real data analysis in the 1000 genomes project, we analyzed all available unrelated samples (n=2460).<br><br>For real data analysis of aDNA, we analyzed all available samples for each of the populations we studied, keeping only the individual with the highest coverage for previously identified families (n=229).
Data exclusions	We excluded related samples based on available data. We excluded samples whose genotype contained more than 75% of missing data.
Replication	We tested our population size inference on 10 independent replicates of each simulation.<br>We compared our results for the analysis of modern DNA with the output of IBDNe
Randomization	Samples were grouped based on their birth location (UK Biobank), population (1kGP) or archeological context (aDNA).
Blinding	We analyzed previously published datasets. There was no blinding involved in our analyses (this is not a controlled randomized study).

Reporting for specific materials, systems and methods

We require information from authors about some types of materials, experimental systems and methods used in many studies. Here, indicate whether each material, system or method listed is relevant to your study. If you are not sure if a list item applies to your research, read the appropriate section before selecting a response.

Materials & experimental systems

n/a	Involved in the study
☒	☐ Antibodies
☒	☐ Eukaryotic cell lines
☒	☐ Palaeontology and archaeology
☒	☐ Animals and other organisms
☒	☐ Clinical data
☒	☐ Dual use research of concern

Methods

n/a	Involved in the study
☒	☐ ChIP-seq
☒	☐ Flow cytometry
☒	☐ MRI-based neuroimaging
